# Friction Stir Welding and Friction Spot Stir Welding Processes of Polymers—State of the Art

**DOI:** 10.3390/ma13102291

**Published:** 2020-05-15

**Authors:** Francesco Lambiase, Hamed Aghajani Derazkola, Abdolreza Simchi

**Affiliations:** 1 Department of Industrial and Information Engineering and Economics, University of L’Aquila, via G. Gronchi 18, Zona Industriale di Pile, 67100 L’Aquila AQ, Italy; 2 Young Researchers and Elites Club, Science and Research Branch, Islamic Azad University, Tehran 14588, Iran; hamed.aghajani@srbiau.ac.ir; 3 Department of Materials Science and Engineering, Sharif University of Technology, P.O. Box 11365-9466, Azadi Avenue, Tehran 14588, Iran; simchi@sharif.edu; 4 Institute for Nanoscience and Nanotechnology, Sharif University of Technology, P.O. Box 11365-9466, Azadi Avenue, Tehran 14588, Iran

**Keywords:** friction stir welding, friction spot stir welding, polymer, state of art, literature review

## Abstract

In the last decade, the friction stir welding of polymers has been increasingly investigated by the means of more and more sophisticated approaches. Since the early studies, which were aimed at proving the feasibility of the process for polymers and identifying suitable processing windows, great improvements have been achieved. This owes to the increasing care of academic researchers and industrial demands. These improvements have their roots in the promising results from pioneer studies; however, they are also the fruits of the adoption of more comprehensive approaches and the multidisciplinary analyses of results. The introduction of instrumented machines has enabled the online measurement of processing loads and temperature, and critical understanding of the principal aspects affecting the material flow and welds quality. Such improvements are also clearly demonstrated by the increase of the strength of recent joints (up to 99% of joining efficiency) as compared to those reached in early researches (almost 47%). This article provides a comprehensive review of the recent progresses on the process fundamentals, quality assessment and the influence of process parameters on the mechanical behavior. In addition, emphasis is given to new developments and future perspectives.

## 1. Introduction

Thermoplastic polymers and reinforced thermoplastics are involved in a growing number of applications owing to their great design flexibility, ease of manufacturing of their complex parts, and their good mechanical strength and high chemical resistance. These aspects have promoted the substitution of metal components with thermoplastics and thermoplastics composites even in structural application fields. An interesting example is represented by the transportation industries, which are engaged with the reduction of vehicle’s weights, with the aim of reducing CO_2_ emissions as well as the increase of the autonomy of electric vehicles. Here, the adoption of fiber-reinforced components for structural parts is growing continuously. These requirements have pushed towards a growing adoption of polymers (and composite materials) due to their ease of manufacturing as well as their high weight-to-strength ratios. Two decades ago, these materials were a prerogative of competition vehicles, such as in Formula 1, or Americas Cup sailboats. Then, fiber reinforced plastics have been applied to fuoriserie vehicles produced by Lamborghini, McLaren, Pagani, etc. Nowadays, many manufactures such as Alfa Romeo, Audi, BMW, etc., (some examples are Alfa Romeo 4C, BMW 7-series and Chevrolet Corvette Z06 [1]) are pursuing the above-mentioned objectives via the greater adoption of reinforced-plastics in structural components, such as the chassis. The greater adoption of thermoplastics is also due to additional advantages including recyclability and reshape capabilities. However, the joining of thermoplastics and fiber-reinforced thermoplastics is still a challenging issue. This paper will provide an overview of the main joining processes that are used to join thermoplastics and reinforced thermoplastics. Common joining techniques such as adhesive bonding and mechanical fastening are discussed first, along with the main welding processes used for thermoplastics. It must be noted that a detailed description of the common joining processes (mechanical joining, adhesive bonding and welding processes of plastic) is beyond the scope of this review. There are many books that describe the specific characteristics of these processes [2,3,4]. Here the main concepts, advantages and limitations are discussed. The aim of this review is to provide the basis for a discussion concerning the state of the art and current trends concerning friction stir welding (FSW) and friction spot stir welding (FSSW) processes as viable alternatives for plastics joining. These processes have been initially developed to overcome the main welding issues of aluminum and its alloys. The great potential of the process has promoted the immediate extension of FSW to various types of metals and metal alloys. Then, FSW and FSSW processes have been applied to thermoplastics. The differences in the rheological behaviors as well as the lower thermal conductivities of polymers (as compared to metals) hinder the possibility to fully inherit the wide knowledge about FSW and FSSW performed on metals. Thus, specific studies were required. Since the first studies concerning friction stir welding applied to polymers [5], growing interest into the possible advantages in this field has attracted the attention of many research groups and industrial manufactures. This has led towards a deeper understanding of the physical phenomena that develop during the process as well as how these phenomena influence the quality of the welds. This knowledge has strongly contributed to process adjustment toward the specific requirements of polymers. Because of the deep differences between polymers and metals, the FSW and FSSW of polymers has required tailored equipment. A comprehensive review of the evolution of the tools adopted for FSW has been reported in [6]; while, a review of FSW of composite structures is reported in [7]. The present paper is aimed at collecting and organizing the main results achieved by available research studies, which have concerned FSW and FSSW processes of polymers. This was done with special attention to the physical and mechanical behavior of polymers that influence these processes when performed on thermoplastics. The aim of the work is to provide a deeper understanding on these processes, and more specifically to describe how the process parameters influence the material flow and how this determines the mechanical behavior of the welds. The paper firstly introduces common joining processes used for thermoplastics, including adhesive bonding, mechanical fastening and plastic welding. Then, the following two sections are dedicated to the friction stir welding and friction spot stir welding processes applied to polymers, with special attention to the influence of the process parameters on the material flow and mechanical behavior. In the last section, the main issue concerning existing literature and future perspectives (also with reference to “Industry 4.0”) are described.

## 2. Common Joining Processes Used for Thermoplastics and Fiber Reinforced Thermoplastics

Common joining processes adopted for thermoplastics and reinforced thermoplastics can be classified into adhesive bonding, mechanical fastening and welding processes. In the following sections, a brief description of these process families is reported with the aim of highlighting the main pro and cons.

### 2.1. Adhesive Bonding

**Figure 1:**
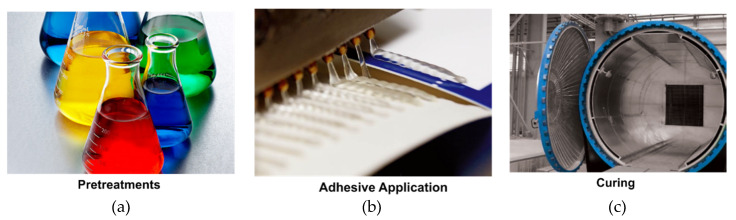
Main phases used in the adhesive binding process. (**a**) Pretreatments, (**b**) Adhesive Application, (**c**) Curing.

Adhesive bonding involves an external adhesive that promotes the formation of weak Van der Waals or even chemical bonding between the adhesive and the two substrates (adherends). In addition, molecular chains entanglements between the adherends and the adhesive may lead to diffusive forces [3]. As the adhesion of the adhesive with the adherends is hindered by trapped air or impurities, the surfaces being coupled are subjected to several pretreatment processes to enable strong and firm connections between the substrates. These generally consist in the mechanical and chemical removal of oil, grease and dust. Surface pre-treatments involve (chemical) solvents for cleaning and degreasing, and (mechanical) treatments such as sandblasting, tumbling and power sanding. In addition, roughing processes are also extensively adopted with the aim of increasing the surface area and to promote regions of mechanical interlock. After these pretreatments, the adhesive is applied over the surfaces of the components to join. Then, a prescribed pressure is applied. The amount of adhesive, the pressure and the coupling time significantly influence the strength of the bonds during service life and must be accurately controlled during this phase. The coupling time is followed by the adhesive curing. When thermosetting polymers are used as adhesive, the curing time enables the crosslinking of the polymeric chains. On the other hand, when thermoplastic polymers are used, the curing phases involve the cooling of the adhesive. Figure 1 schematizes the three phases for adhesive bonding.

**Figure 2:**
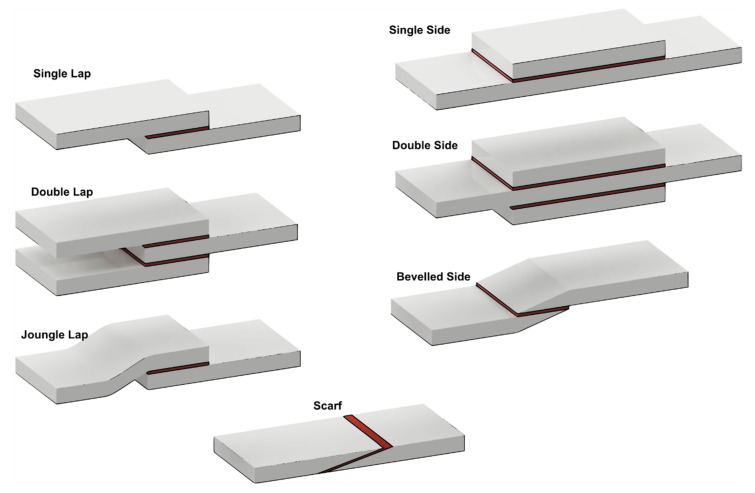
Typical sheets configurations used in adhesive bonding.

Adhesive bonds generally show greater shear strength as compared to tensile strength. Thus, the joint profile is designed with the aim of promoting shear forces. Figure 2 illustrates different bond layouts commonly involved. These differences are aimed to promote specific features. For example, the adoption of double lap and double side configurations enable the promotion of the load bearing capability by increasing the adhesion surface, as compared to the single lap and single side, respectively. On the other hand, other configurations are more aimed at achieving some geometrical characteristics. For example, the joungle lap enables the collinearity of the external surface, while the beveled side enables a uniform sheet thickness even along the bonded region. Finally, the scarf configuration enables an almost invisible bond as both the surfaces of the two components coincide. However, these configurations require the edge of the sheets to be machined. As a result, adhesive bonding is characterized by several advantages such as the possibility of joining different materials, including thermoplastics, thermosets, metals, ceramic and wood. Adhesive bonding enables continuous connections, low weight increase (as the adhesive layer is relatively thin: 0.05–1 mm), sealing of the substrates, as well as the possibility to prevent from metals corrosion as the electrical insulating behavior. On the other hand, adhesives can operate in a limited range of temperatures. Some adhesive become extremely brittle at low temperature while degradation may occur at high temperatures. Rigorous and lengthy preparation of the adherends surfaces is required in order to enable a stable bonding between the adherends and the adhesive. In addition, adhesive bonds are affected by great uncertainty concerning long-duration resistance and environmental sensitivity. The process has a great environmental impact, as the adoption of solvents during the surfaces’ pretreatment and production creates hazardous vapors during curing. This environmental impact is also affected by the production of volatile solvents used during pretreatments. Finally, disassembly is not generally possible without the destruction of the components involved.

### 2.2. Mechanical Fastening

**Figure 3:**
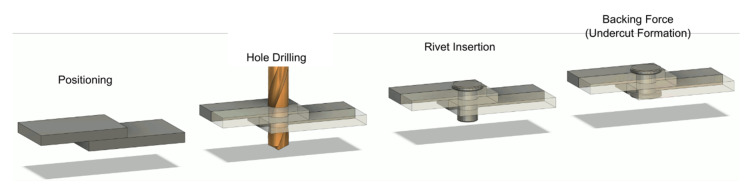
Main phases of riveting.

Mechanical joining processes generally involve external fasteners. These are used for the formation of mechanical interlocks between the components and commonly require predrilled holes whereas the fasteners are inserted. For example, when rivets are used, the flanges/sheets being joined are accurately positioned, then a pass-through hole is drilled through both the components, then the rivet is inserted, and the second undercut is produced. A schematic of the main phases required for drilling is reported in Figure 3.

Thus, the joining time is much shorter than adhesive bonding. Nevertheless, especially for applications where numerous joints must be produced, some process modifications can be adopted to further reduce the joining time, such as the adoption of self-pierce rivets, or mechanical clinching. In self-pierce riveting, the rivet is directly punched throughout the (overlapping) sheets. The rivets involved are characterized by a special geometry that enables to punch-out the upper sheet and then produce an interlock between the upper and bottom sheets. Mechanical clinching produces an interlock between the sheet by means of a material flow induced by a punch and a die. Thus, the process does not involve external fasteners with evident advantages in terms of weighting of the structure and costs. However, mechanical clinching is more suitable to produce hybrid metal-polymer connections rather than polymer–polymer ones. Actually, despite a certain number of scientific works concerning the clinching of hybrid connections involving polymers or reinforced polymers [8,9,10,11], so far any experimental evidence for the feasibility of clinching has not been provided concerning polymer joining. Mechanical fastening can be performed by the adoption of external fastener, direct molding of connector elements (such as snap-fits) as well as thermoforming of the plastics regions, as summarized in Table 1.

Mechanical joining processes, with few exceptions (e.g., hemming) involve spot joints. This comes with high stress concentration, and in the case of reinforced plastics, it also may come with fiber interruption. In addition, the employment of external fasteners comes with an increase in weight as well as costs increase. Finally, because the joining nature of these connections requires the formation of an undercut, mechanical connections are generally hindered to lap configuration (butt joints are not possible). The main advantages and disadvantages of mechanical fastening are summarized in Table 2.

### 2.3. Plastics Welding

Plastic welding processes can be adopted to join thermoplastic polymers. Plastics welding is based on the formation of molecular bonds between the materials. This requires the material to be heated above the softening/melting point. This enables a certain mobility of the polymer chains. Then, an external pressure is applied to produce molecular bonds, followed by a cooling phase. A detailed description of plastic welding can be found in [3]. Most of the welding processes performed on plastics involve heating of the interface over the softening/melting point of the materials and then pressing together the components. Thus, the components weld upon enough cooling. Different methods have been developed using different heat sources, which can be classified in:

- Conduction heating: the heat is conducted from an external tool. Typical examples include heated tool welding, plate welding and resistive implant welding;
- Friction welding: the heat is generated by bringing into contact two workpieces under pressure, and a reciprocating motion (vibration) is applied along the interface. Here, the friction induces the increase in temperature of the material close to the components interface. Typical examples are vibration welding, ultrasonic welding;
- Wave induced welding: here, the materials are subjected to external waves (e.g., electromagnetic field). For example, radiofrequency welding involves high frequency (commonly 27.12 MHz) electromagnetic energy that is coupled with the material leading to a conversion of alternating electric energy to heat. Another example is represented by laser welding. Here, the optical coupling between a laser source and the polymeric materials is exploited instead of electric coupling. Examples include infrared welding, induction welding and microwave welding.
- Convective heating: the weld seam is heated by means of a convective source (typically a hot gas) e.g., hot gas welding;
- Heated filler: these processes involve an external (polymeric) filler that is previously heated and pressed against the two materials along the weld seam (e.g., extrusion welding).

## 3. Friction Stir Welding

Friction stir welding is a thermo-mechanical process involving a non-consumable rotating tool that locally softens the underlying material through heat produced by frictional and plastic work. This enables the softened material to be stirred leading to the weld achievement [12]. The process was patented in 1991 by The Welding Institute (TWI) for the joining of aluminum alloys [13]. As the frictional heat depends on contact pressure exerted by the material on the tool, when the material temperature rises, the frictional heat reduces. This reduces the possibility to achieve material melting during the process, and consequently avoids many issues including change in state, changes in gas solubility and volumetric changes, which often affect fusion welding processes. As the lower temperature is reached during FSW, the process also enables lower material distortion and residual stress, leading to improved fatigue life, even on extremely thin and thick components. Nevertheless, as there are heavy mechanical forces during the process, so the components must be firmly clamped in order to prevent possible displacements. In addition, FSW demands high performing machines leading to high processing costs (as compared to typical welding processes). However, this also comes with higher process reliability, robustness, standardization and lower operator skills. In addition, FSW enables lower production of hazardous fumes, and requires lower energy that result in minor environmental impact. As the stirring material is under a “pasty” state (the material melts rarely), FSW offers higher flexibility concerning the orientation of the weld path, as it is not affected by gravitational effects. The main pros and cons of FSW process are summarized in Table 3.

A key aspect determining the quality of the welds is the design of the welding tool. Despite the development of many alternatives, the tool generally consists of a shoulder and a pin. The shoulder slides on the surface of the sheets; on the other hand, the tool pin (with a diameter smaller than the shoulder) almost completely penetrates the sheets (the pin length is generally 90% of the sheet thickness). The pin is designed to soften and stir the material to ensure the complete penetration of the weld through the thickness of the weld line. The tool shoulder is designed to prevent the material ejection from the weld line and even to preheat the material being stirred. The sheets can be arranged according to different configurations including butt, lap and T-shaped joints.

**Figure 4:**
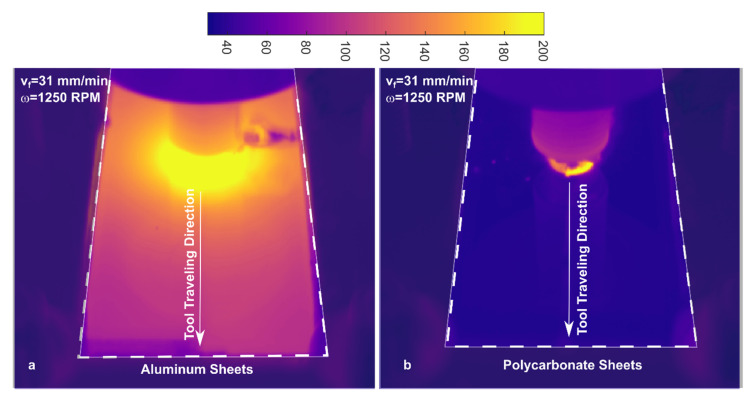
Temperature distribution during FSW of: (**a**) aluminum [14] and (**b**) polycarbonate [15].

During FSW, the friction at the tool/material interface produces a great amount of heat that produces high rise of the material temperature. In metals, the heat is immediately spread towards regions surrounding the tool, leading to a “diffused heating”, as reported in [14] and shown in Figure 4a. For polymeric materials, which are characterized by much lower thermal diffusivity as compared to metals, the heated region is highly confined within the weld seam, as shown in Figure 4b.

In polymers, as the material being stirred exceeds the glass temperature, the polymeric chains become more and more free to flow. Under these conditions, the stirring effect can develop even at the molecular scale. The polymeric chains of the two components mingle if the materials cool down the T_g_ of the polymer. Because of the high pressure (in the stirred region) and high temperatures, typical welding conditions may occur during the process. However, it is worth noting that, the main bonding mechanism is represented by the (mechanical) stirring effect. This enables the feasibility of the process for hybrid connections made on different materials, even metals and polymers [12,16]. The main limitations on the applicability of FSW are, theoretically, the capability of the material to flow (at the process temperature).

The deep differences between polymers and metals produce the different thermal distributions shown in Figure 4, and come with additional issues, including:

- Lower material preheating (ahead the tools shoulder) owing to low thermal diffusivity of polymers;
- Longer cooling time due to lower heat diffusion towards the surrounding material once the tool has passed by a given position;
- In polymers, moisture content may give rise to bubble development, which affects the mechanical behavior of the welds;
- Severe reduction of the load bearing capacity (especially in semi-crystalline polymers) as the softening/melting temperature is approached. This may lead to unsteady material flow conditions;
- Polymeric chains have different lengths; thus, melting conditions may occur at some regions (where shorter chains are localized) leading to uneven material flow;
- Because of the poor thermal diffusivity of the polymers, the material under the tool shoulder is rapidly heated even above the softening/melting point (owing to the high tangential speed at the interface). This would cause the material ejection from the weld seam, with consequent reduction of the strength of the welds. Thus, FSW of polymer materials is preferably performed with a non-rotating tool shoulder;
- The poor thermal diffusivity of polymers also comes with steeper temperature gradients between the stirred and the surrounding regions. This may cause poor adhesion at the interface and differential shrinkage. In addition, the interface between these regions is also affected by the presence of porosities (often generated by the presence of moisture), that act as stress raiser. As a result, the interface between the stirred region and base material is even more critical in polymers than in welds performed in metals.

### 3.1. Description of the Main Phases

Linear FSW involves a sequence of different phases [17] including:

- Plunge phase: During this phase, the rotational tool moves in axial direction and plunged the cold materials. Downward motion of the tool is stopped as soon as the tool shoulder is in contact with the upper surface of the workpiece. In this phase, the axial force and the torque applied to the linear FSW (LFSW) tool reach a peak value and immediately reduce as the steep material heating. At the end of this phase, the LFSW tool pin was fully surrounded/embedded by the material to be stirred. The depth by which the tool shoulder penetrated the upper sheet surface is called plunge depth (TPD) [18].
- Dwell (stabilization) phase: At the end of the plunging phase, the tool is held at a prescribed position leading to material preheating;
- Advancing (welding) phase: The LFSW tool starts to move forward along weld seam. The tool velocity (V) in this phase represents the key aspect that determines the quality of the weld. Depending on the LFSW tool design, V and TPD, the LFSW tool may be tilted to improve the quality of the joint line.
- LFSW tool retract phase: As the tool reached the final position, the tool is rapidly removed from the weld seam [19].

**Figure 5:**
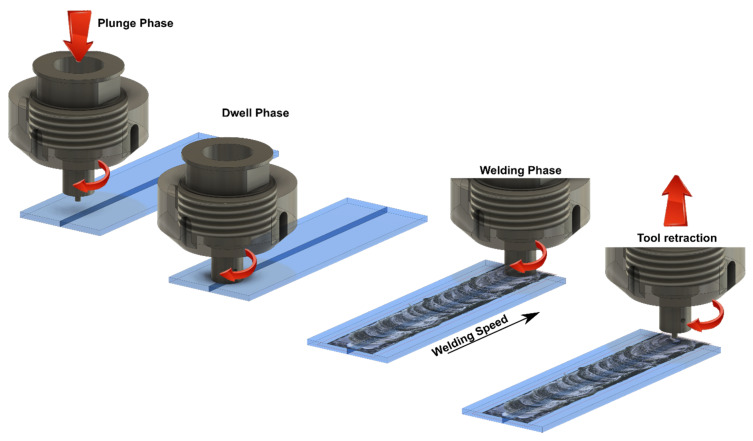
Main phases of FSW: plunging, dwell, welding and tool retraction.

Figure 5 shows a schematic of main phases of FSW.

### 3.2. Material Flow

**Figure 6:**
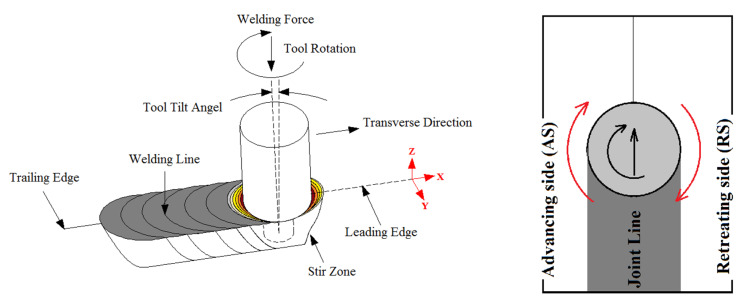
Linear friction stir welding illustration and different parts name [20].

During the process, the tool extrudes the preheated material from the leading edge of the weld seam into the stir zone (SZ). The applied force for material flow is highly affected by tool tilt angle (TTA), which is an angle with negative position from forward transverse direction [20,21]. After passing the tool from SZ, the axial force causes the extruded material to fill the weld seam [22]. Three-dimensional observation of the joint area has indicated that the cross section consists of 4 main regions [23], that are schematically shown in Figure 6:

- Stir zone (SZ): The center of weld seam where the material undergoes to high rate of thermal and mechanical stirring cycles.
- Thermo-mechanical affected zone (TMAZ): A narrow area around SZ where the material experiences low rate of thermal and mechanical stirring cycles.
- Heat affected zone (HAZ): The region surrounding the TMAZ where the material undergoes thermal cycles diffused from SZ.
- Base material (BS): The neat area where the material is not involved into thermal and mechanical cycles.

**Figure 7:**
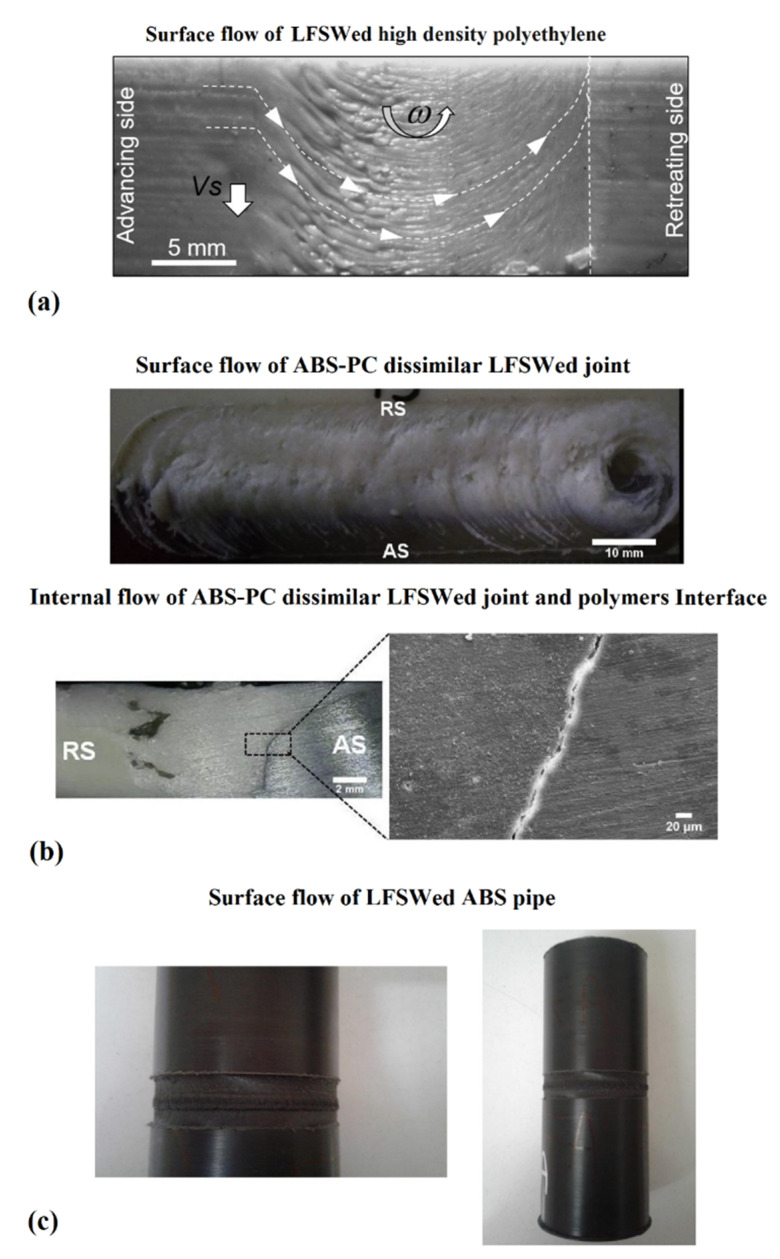
(**a**) Surface flow of high-density polyethylene (HDPE) joint after linear FSW (LFSW) [25], (**b**) surface and internal flow of ABS/PC dissimilar LFSW joint [27] and (**c**) digital image of HDPE pipe joint after LFSW [29].

Formation of voids, flashes, and defects on the surface and internal parts of the joints are important issues in LFSW of polymers [20]. Micro and macro observations of the weld seam are indicative of the material flow produced [24]. For transparent polymers, a virtual measuring machine (VMM) can be employed while for opaque polymers, optical microscopes are desired to study the material flow [25]. Figure 7a shows the material flow produced during FSW of high-density polyethylene (HDPE). The results reported in [26] highlighted the existence of a relationship between revolution pitch (R) and weld surface appearance. Particularly, the study revealed that a decrease of R-value produced a smoother weld surface with fewer irregularities. FSW is characterized by an asymmetrical distribution of the temperature at the advancing and retracting side. This is mainly due to the change of direction of the tangential speed on the tool. Thus, at the advancing side (AS), the tool-material relative speed is given by the sum of the welding speed plus the tangential speed (of the tool). On the other hand, at the retracting side (RS), the relative velocity is given by the difference of the welding speed and the tangential speed of the tool. This causes more heat produced at the advancing side with respect to that produced at the retracting side. Therefore, the AS receives more heat than the RS, leading to lower cohesion in the RS, and molecular structure orientation is more affected [26]. The mechanism of material flow in dissimilar polymeric joints are different and attaining sound joints is still a challenging issue. Kumar and Roy [27] employed a double-step shoulder tool with a right-angle threated pin profile to join Arilonitrile Butadiene Styrene ABS (set at retreating side) to polycarbonate PC (set at advancing side). Figure 7b shows the formation of a sound and defect-free weld line in ABS/PC joints. The applied downward force and the adoption of a double shoulder and threaded pin enabled an appropriate material flow leading to the achievement of sound welds. Thermoplastic polymers are characterized by high sensitivity by temperature. This also induces great temperature-dependent mechanical behavior variations. This is particularly important when characteristic temperatures of the polymer (such as glass transition, softening/melting points) are reached. The generated heat should not be too high to cause excessive melting of the polymers expelling the softened materials in the outward direction from the weld line [27], leading to the formation of root defect. This is also straightly related to the LFSW pin length, as reported in [28]. Arici et al. [28] proved the suitability of double pass LFSW on polyethylene (PE) sheets with the aim of eliminating the root defect.

Despite that the linear FSW of flat sheets has been attracting the interest of the greater portion of the studies, the suitability of the process for the connection of the pipelines is increasing. Here the main issue is represented by the presence of voids on the surface, as the curved surface of the pipes. An example of the successful joining of HDPE pipes by LFSW process is shown in Figure 7c. Herein, a stepwise setup was designed achieve high-quality LFSW joint [29]. However, a deep understanding about LFSW of curved polymeric materials is still missing.

### 3.3. Morphology of the Welds and Quality Assessment

Different characterization methods can be performed with the aim of deeply analyzing the quality of the welds. Quality assessment generally involve mechanical characterization of the welds. However, as steep thermal cycles develop during the FSW process, thermal modification of the polymer may also influence the quality of the welds. The main mechanical testing methods used for polymeric materials are tensile and bending (flexural) tests in accordance with ASTM D638 standard [30], which is different than that of metallic materials [24]. To determine the energy absorption, an impact test is usually performed according to ASTM D256 standard [31].

**Figure 8:**
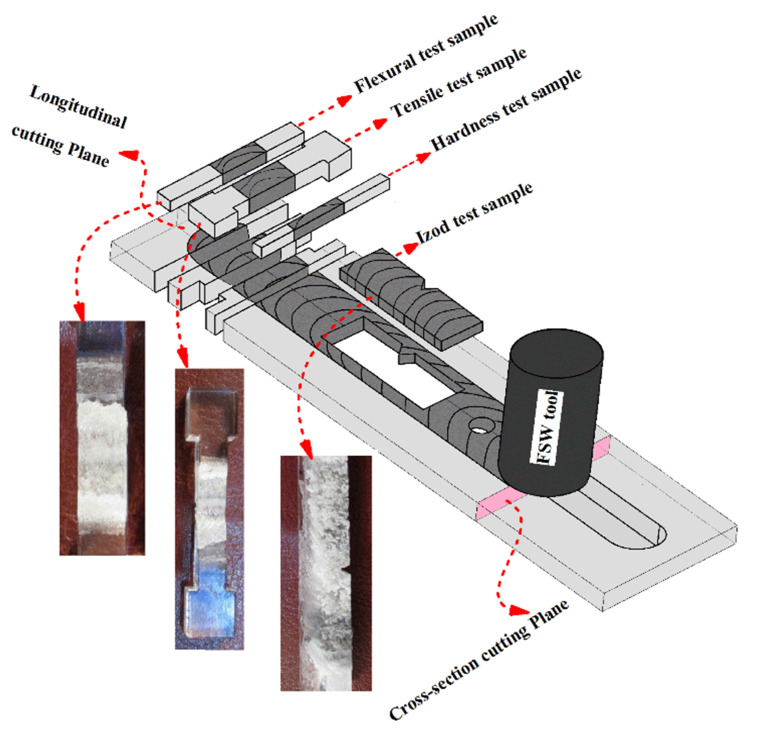
Linear friction stir welding test sample [24].

The above characterization methods provide averaged measurements of the strain. On the other hand, to better understand the influence of the process parameters on the weld quality, local properties would be more appropriate. This has been done by the adoption of hardness and micro-hardness analysis as well as digital image correlation (DIC) techniques. Changes in the hardness in the joint line are determined in ShoreD scale [32] based on ASTM D2240 standard [33]. Other methods of hardness testing such as Brinell may be used to measure the hardness changes in FSW of hard polymeric materials [31]. In addition, LFSW increases the planar anisotropy. Thus, mechanical tests should be performed in both longitudinal and traverse to the weld line. Since the length of the welds, different samples can be extracted from a weld enabling a comprehensive analysis of the welds. Figure 8 shows the method of sample preparation for mechanical testing of LFSWed polymers.

**Figure 9:**
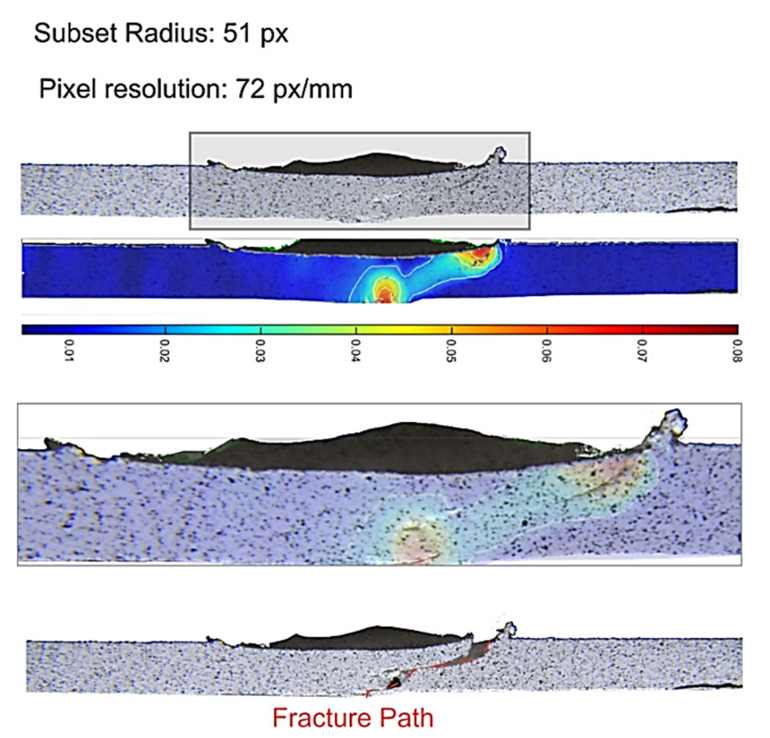
Details of prepared samples for digital image correlation (DIC) analysis during tensile tests [34].

Strain changes during the tensile test can be monitored by digital image correlation (DIC) analysis [34]. Lambiase et al. [34] used DIC analysis to show the strain development during tensile tests of FSWed PC sample (Figure 9). They analyzed samples were FSWed with various tool velocities, and found that at the beginning of the tensile test the strain localized in the region corresponding to the contact with the tool shoulder corner. Linear friction stir welding shares and inherits some features from extrusion welding. In LFSW the extruded polymer is substituted with the stirred material that is highly pasty and must fulfill the weld seam. This is done by the tool shoulder (despite the hot shoe used in extrusion welding), which exerts an upsetting force on the stirred material. Consequently, the stirred material is forced to adhere to the surrounding walls of the weld seam. The morphology of the stirred region (shape, dimension and presence of cavities), the adhesion of the stirred region to the surrounding walls, as well as the thermal shrinkage determine the mechanical behavior of the welds. Lambiase et al. [34] collected three main failure modes developing during tensile tests of welds made by FSW, namely:

- Adhesive failure: detachment of the stirred region from the side walls. This failure mode mainly developed due to low temperature and hydrostatic stress developed during the process;
- Cohesive failure: failure developing from the weld seam due to the presence of defects such as tunneling and/or porosities;
- Stress concentration: due to the material removal from the upper weld surface due to adhesion of the material to the bottom surface of the tool shoulder, which exerted a milling action.

**Figure 10:**
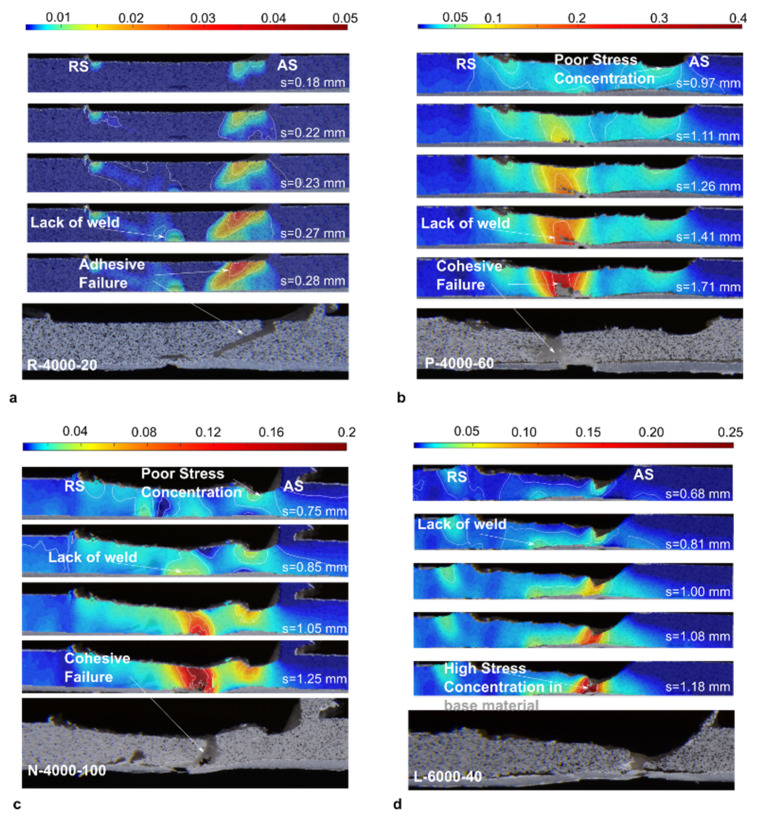
Failure modes and relative strain maps determined by means of DIC analysis [34]. (**a**) Adhesive failure; (**b**,**c**) cohesive failures and (**d**) stress concentration.

These failure modes were determined with the aid of DIC analysis as reported in Figure 10. During stir welding, the frictional heat increases the local temperature. This may lead to changes in the molecular structure of thermoplastics [7]. Differential scanning calorimetry (DSC) and thermogravimetric analysis (TGA) are effective methods to examine the possible structural changes through thermal analysis [34]. Changes in thermal properties could be attributed to thermal or oxidative degradation or variation in the degree of crystallinity of the polymer [7]. Phase transitions, chemical reactions, vaporization, sublimation and desorption of elements can also affect the thermal properties [35,36]. ASTM D3418–15 standard [7] describes the method of DSC examination of polymers.

**Figure 11:**
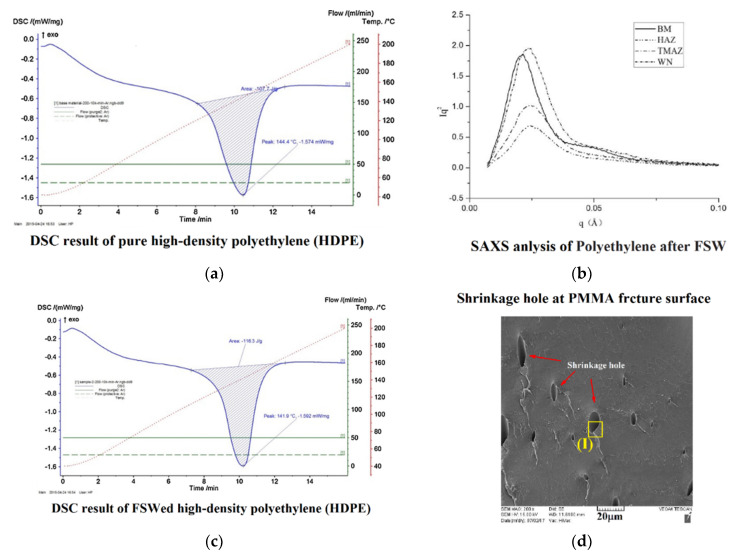
Differential scanning calorimetry (DSC) results of HDPE before (**a**) and after (**b**) LFSW [36], (**c**) small angle X-ray scattering (SAXS) analysis of polyethylene (PE) [37] and (**d**) shrinkage holes of PMMA on fracture surface of tensile sample [25].

Some researchers also used X-ray analysis including X-Ray diffraction (XRD) and small angle X-ray scattering (SAXS) to determine the crystal orientation of polymers the LFSW process [37]. Representative results of DSC and SAXS for LFSWed HDPE are shown in Figure 11a–c. Due to the high strain rate of the polymers during the stirring action and after cooling, shrinkage voids are formed. As the heat input increases, the peak temperature increases thus affecting the crystallinity and molecular weight of the processed polymer [7]. Consequently, the amount of shrinkage increases to form more and bigger pores in the Stirred Zone SZ [24]. The formation of shrinkage holes decreases the mechanical strength of the welded polymer. They involve a reduction of the cross-sectional area and act as stress raiser. The shrinkage voids are detectable in the fracture surface of the samples, as shown in Figure 11d for tensile tested Poly(methyl methacrylate) PMMA.

### 3.4. Effects of Processing Parameters on the Quality of Polymer Joints

The main FSW process parameters are mechanical parameters that exerted by LFSW tool on joint line. To better understanding the effects of the LFSW process parameters on the properties of polymeric material joints, the LFSW process parameters are summarized in Table 4.

**Figure 12:**
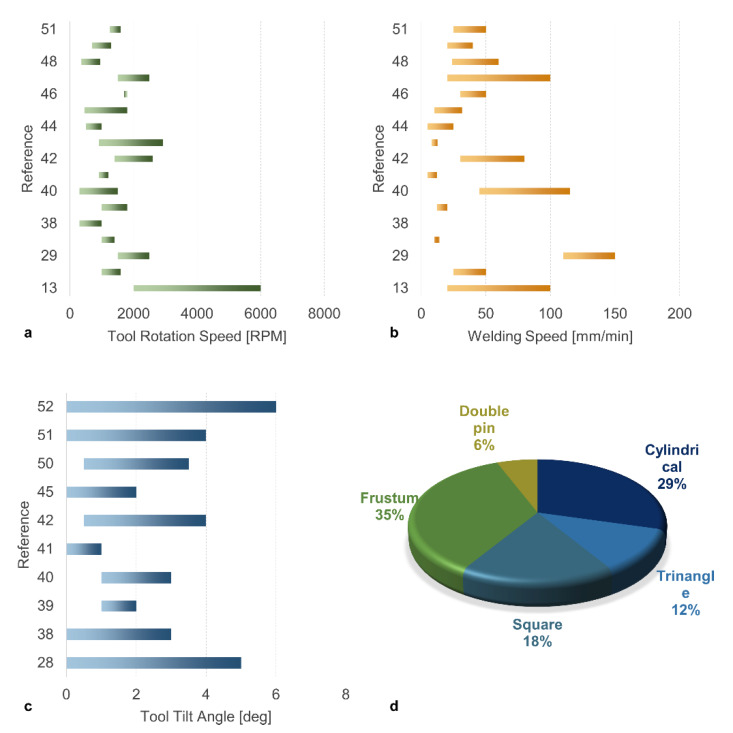
Processing windows analyzed in different papers concerning FSSW of polymers. Effect of: (**a**) tool rotation speed; (**b**) welding speed; (**c**) tool tilt angle (**d**) shape of pin.

A graphical representation of the ranges analyzed in different references for the tool rotation speed, welding speed, tool tilt angle and pin geometry is depicted in Figure 12. The comparison in Figure 12a indicates a general consistency concerning the tool rotation speed as most of the studies focused the range 1000–2000 RPM. Similarly, a general consistency concerning the tool tilt angle was found; in most of the studies the range between 1–3 degrees was investigated, and it was studied up to values of 6 degrees, as shown in Figure 12c. On the other hand, higher heterogeneity was found concerning the welding speed. Indeed, two separate ranges can be found in Figure 12b: a low welding speed range (10–30 mm/min) and high range (higher than 30 mm/min and up to 150 mm/min). Figure 12d compares the tool pin shape. The distribution indicates that, frustum and cylindrical pin were the most studied shapes for the pin (almost 65% of the studies involved these pin shapes), while the remaining 35% of the works adopted different tools such as square, triangle and double pin shapes.

### 3.5. Rotation Speed

**Figure 13:**
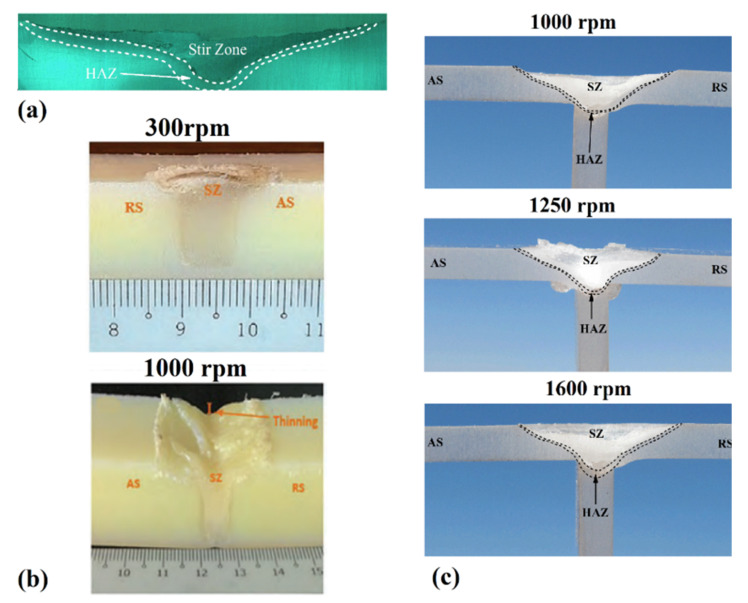
(**a**) Internal flow of PMMA [51], and effects of ω on (**b**) internal flow of Nylon 6 lap joint [38] and (**c**) PMMA T-joint [25].

The rotation speed ω directly influences the amount of frictional heat produced. Due to the nature of FSW process as a semi-solid-state joining process, the temperature at SZ generally does not exceed 85–95% of the melting point of the base polymer. Notably and unlike metallic materials, TMAZ is rarely seen in polymeric welded joints [25]. With increasing ω, the size of the SZ increases as higher amount of heat is supplied. Similarly, it also influences the dimension of the HAZ as reported in [51,53]. The experimental findings indicated that in LFSW of PMMA, the size of HAZ increases with increasing ω. The flowability of PMMA increased at higher ω values while the distance between surface flow rings decreased. Despite benefits of higher ω for flow of PMMA, uncontrolled ω at very high rotation speed caused materials ejection from the welds seam [25]. Under high rotation speeds, material flash also appeared as the excess of semi-molten polymer squeezed out of SZ [38]. The material ejected induced a thinning of the weld seam (Figure 13b) that affects the tensile strength. The appropriate selection of the rotation speed that directly influences the temperature of the material being stirred is fundamental. Controlling the rotation speed enabled a strength of LFSWed in polyethylene sheets of almost 75% of the base material [39]. Bozkurt [40] employed L9 Taguchi orthogonal design of experiments to demonstrate the importance of rotational velocity on the strength of high-density polyethylene (HDPE). It was found that ω was the main contributor in the mechanical strength of the joints. Conversely, when incorrect and low rotational velocity is used, the insufficient heat generated does not soften the material enough to produce a strong and sound weldment. These conditions lead to weak bonding of the stirred region with surrounding region. Conversely, high rotational velocities involve the ejection of the material from the butt line as the low viscosity of the polymer.

### 3.6. Welding Speed

**Figure 14:**
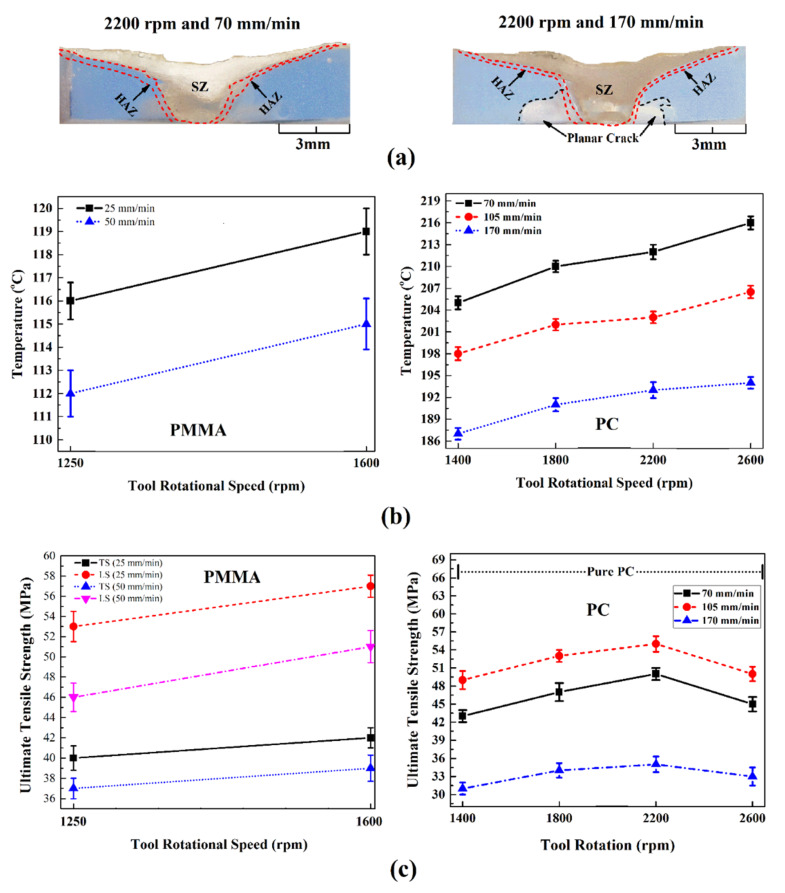
(**a**) effects of welding speed (V) on internal flow of polycarbonate (PC) joint after LFSW [42], (**b**) effects of welding speed on maximum recorded heat during LFSW of PC and PMMA [42,51], (**c**) effects of welding speed on tensile strength of PC and PMMA after LFSW [42,51].

The peak temperature during LFSW of polymers depends on the welding speed (V). High welding speed involves lower interaction time. This induces lower frictional heat diffusion in SZ [41]. On the other, the reduced frictional heat diffusion at leading led to the formation of uncompleted SZ in the weld seam. This phenomenon is one of the challenging issues in FSW of polymeric materials [54]. During joining of some polymers, high welding speed caused formation of cracks at root of SZ [25]. Due to low thermal diffusivity of thermoplastics, the preheated zone in leading edge is much smaller than that observed on metals. Inversely, at high welding speeds, the preheating effect is negligible, and the tool enters in contact with a “cold” material leading to the development of tiny cracks. These cracks grow to form planar cracks around the weld seam and at the root of SZ [42], as shown in Figure 14 for polycarbonate (PC).

Studies of FSW of ultra-high molecular weight polyethylene (UHMW-PE) indicated that when high welding speed were adopted, the plastic material was cast away from the pin edges before enough heat was produced leading to unsuccessful joining. Thus, gradual pre-heating (by reducing V) at the leading edge of UHMW-PE was required to obtain defect-free joints. As the welding speed directly affects the material flow and formation of defects, the mechanical properties of FSWed polymers was significantly influenced by this processing parameter. For instance, Figure 14 shows the effect of V on the peak temperature and mechanical properties of PC and PMMA. Increasing the heat input, the tensile strength of the weldment in longitudinal (LS) and traverse (TS) directions was increased. With increasing ω and decreasing V, the mechanical strength reached a maximum value at intermediate conditions.

### 3.7. Tool Tilt Angle (TTA)

Positive tool tilt angle increases the extrusion force on polymeric materials from leading edge to trailing edge [42]. TTA directly affects the flow of the plasticized material around the shoulder [21]. With increasing TTA, more downward forging force is applied and more frictional heat is produced [16]. TTA improves the material flow from the front to the back side of the tool leading to a better filling of SZ. TTA also influences the amount of generated heat. Low TTA forms voids in the SZ root and similarly, voids may be formed at high angles. Generally, in LFSW of polymeric materials, the tilt angle affects the shape of the SZ. Saeedy and Besharati Givi [39] have shown that high TTAs involve several defects, especially tunneling ones. High TTAs involve greater thinning of the SR leading to a significant reduction of the tensile strength of the welds [55].

**Figure 15:**
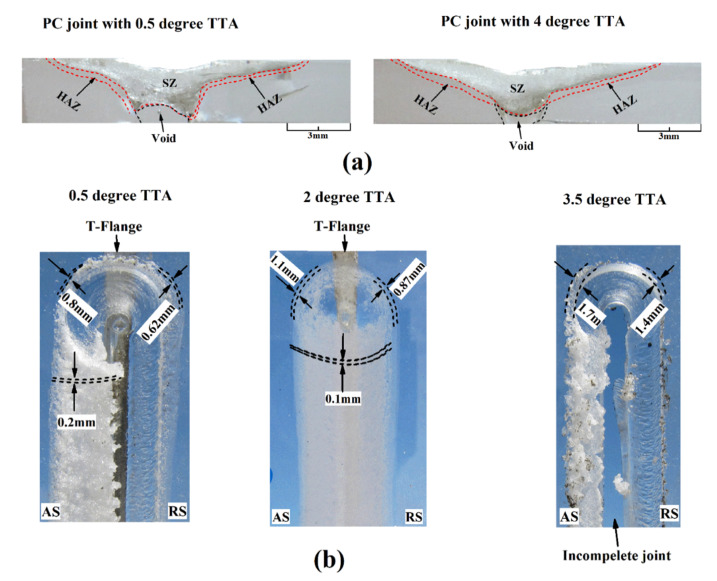
(**a**) Effects of tool tilt angle (TTA) on internal flow of PC joint after LFSW [51], (**b**) effects of LFSW tool tilt angle on surface flow of PMMA T-joint [56].

The effect of TTA on the generated heat and defects formation in PC was also investigated in [42]. The results indicated that an inappropriate TTA forms root tunnel voids in SZ and affects the shape of SZ. At low tilt angles, convex shape SZ is formed while at high angles a concave shape at the base of the weld root is seen. The effects were related to the peak temperature and the material flow during LFSW (Figure 15a). Lambiase et al. [50] reported that during LFSW of PC, TTA higher than 2o led to great amount of material ejection from the seam line and formed a coherent side flash. The formed flash worsened the surface appearance of the weld line and required subsequent machining. In addition, this originated the tunneling defect that severely affects the weld strength. Similar results on PMMA revealed that with increasing TTA more downward forging force is applied leading to higher frictional heat. Extreme TTAs may also lead to complete material ejection from the weld seam [51]. Wrinkles and surface voids may also be formed on the upper surface (Figure 15b).

### 3.8. Tool Plunge Depth (TPD)

**Figure 16:**
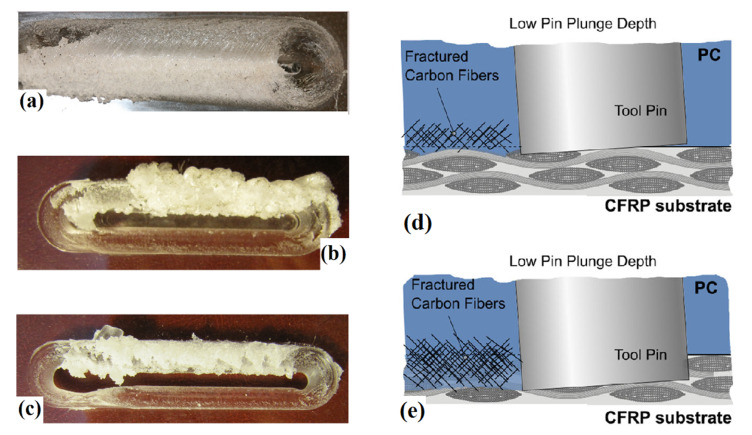
Digital images show the material flow at the surface of PMMA weldments processed at plunge depths of (**a**) 0.2, (**b**) 0.4, and (**c**) 0.6 mm [51]. Effects of (**d**) low tool plunge depth (TPD) and (**e**) high TPD on carbon fiber array changes at interface of PC and CFRP [56].

The tool plunge depth (TPD) influences the amount of frictional heat and interactions between the tool and the polymer (i.e., sliding and sticking). It also affects the axial/welding force at the tool-sheet interface [42]. The work indicated that higher TPD led to higher peak temperature of the material (PC) as well as larger SZ. However, at high TPDs, large flashes were produced in the AS. Similar results have been reported for PMMA, but the heat was more distributed in AS as compared to RS [51]. At very high TPDs, the tool shoulder deeply penetrated the PMMA and tool body hinders the material flow from AS into RS (Figure 16). This phenomenon led to sticking of PMMA under the tool shoulder surface. As a result of the higher axial force at the high plunge depth, the plasticized material was pushed towards out-of-stir zone and a long duct was formed. At an optimum level of TPD, the tensile strength becomes maximum. However, the highest strength is achieved in LS, which is attributed to the formation of the tissue-like structure that makes tensile sample stronger at filament path than at woof direction. The effects of TPD on dissimilar lap joints made of PC and carbon fiber-reinforced plastic (CFRP) revealed that mechanical properties of joint can be changed by altering of pin TPD at interface of base materials [56].

Here, the PC placed at top and the results indicated that higher TPD produced fiber interruption, presence of tunneling defect, and stress concentration which yielded to lower strength and absorbed energy (Figure 16). On the other hand, the joints made with lower TPD showed higher strength but lower absorbed energy. This was due to a higher viscosity of the polycarbonate passing throughout the carbon fibers that slightly replaced the underlying epoxy.

### 3.9. Probe Shape

**Figure 17:**
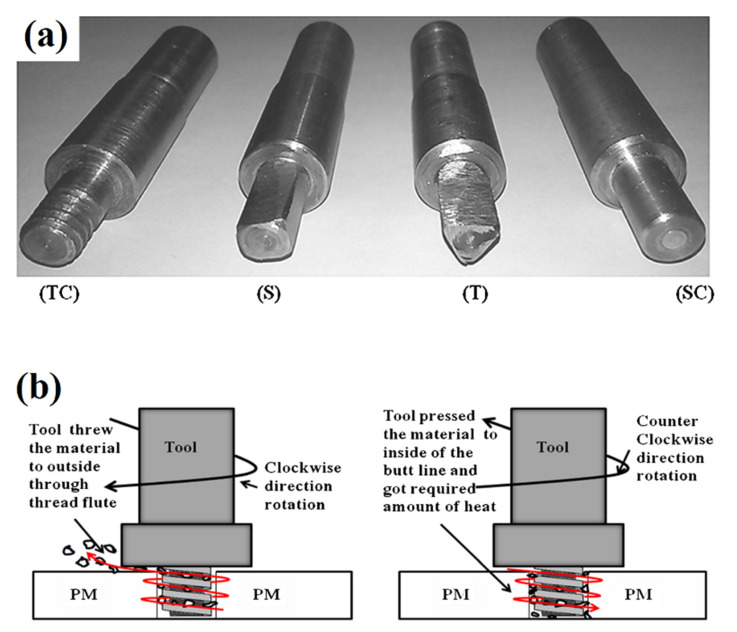
Different pin profile that used for LFSW of (**a**) polypropylene PP, polyethylene PE [43] and (**b**) ABS [57].

The shape of the FSW tool is one of the most important factors affecting the internal and external flow of polymeric materials and consequently mechanical properties of FSW joints [30]. Hajideh et al. [43] studied the effect of pin profiles on LFSW of PE and PP sheets. Four tools profiles with square (S), threaded cylindrical (TC), triangular (T), and straight cylindrical (SC) shapes were used (Figure 17). The pin with the threaded cylindrical tool yielded stronger joints while the straight cylindrical pin produced the weakest ones. Although tools with squared and triangular shapes stir the plasticized material at the SZ more severely, the joint strength of the specimens prepared by threaded cylindrical tool was higher. Sadeghian and Besharati Givi [57] reported that the material stirring during FSW of Acrylonitrile Butadiene Styrene (ABS) with conical pin tool was more uniform than cylindrical pin. Panneerselvam and Lenin [58] reported that FSW of PA6 by threaded pin with rotation in counter clockwise direction was efficient leading to formation of sound SZ with high mechanical behavior (Figure 10b). The results reported by Sahu et al. [55] indicated an increase of the strength of the welds made with a square tool. This was addressed to an increase of the heat produced, which improved the flow of the plasticized material around the tool. Moreover, the pin generates a pulsating action because of the flat faces, which helps the material stirring. Conversely, cylindrical pins produce a weaker stirring effect of softened thermoplastic materials. Hoseinlaghab et al. [45] reported that the effect of tool pin profile on FSW of HDPE was relatively independent to the welding parameters. However, the creep strength of HDPE after FSW with cylindrical pins was lower than the conical profile.

### 3.10. Environment

**Figure 18:**
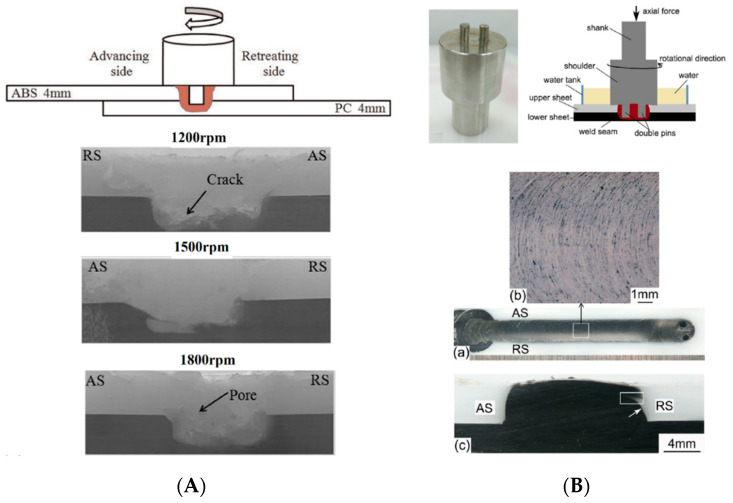
(**A**) Effect of process parameters on underwater LFSW of ABS-PC dissimilar joint [59] and (**B**) effect of the double pin in underwater FSW of HDPE sheets [60].

LFSW of polymers under different environments can change the quality and properties of the welds. Unfortunately, no comprehensive studies on LFSW of polymeric materials under various environments such as vacuum or underwater are available in open literature. Gao et al. [59] investigated underwater LFSW of dissimilar joints between ABS and PC. Various range of welding parameters (ω, V and TPD) were studied to determine the suitable working condition to attain a sound joint with high tensile (Figure 18A). The highest tensile strength (19.2 MPa) was achieved at 1500 rpm, 40 mm/min welding speed, and 1.0 mm tool plunge depth. Trapped air and shrinkage pores were the main defects. Other studies on HDPE [37,60] determined that the tool pin profile exerts great influence on the successful underwater LFSW. It was shown that the surface flow of underwater joints was improved by employing double pin profile. The flash formation at surface of HDPE joint was also removed.

### 3.11. Process Monitoring

Monitoring of the LFSW process can provide great insight concerning the process development as well as the development of abnormal conditions. Process monitoring enables deeper process understanding, especially to provide a fully comprehension of the influence of the process parameters on the weld’s quality and onset of defects. In addition, it is the basis for “Intelligent Machines” development, which are capable to adjust the processing conditions during the process with the aim of increasing productivity, energy efficiency, and improving the quality of the welds for example by hindering processing conditions that lead to occurrence of defects. Process monitoring is generally performed by means of load and temperature measurement equipment. During load measurements, the bottom plate, which supports the sheets being welded, is fixed to a piezoelectric transducer and a charge amplifier [17]. Otherwise, the load can be measured by more expensive equipment directly mounted between the mandrel and the tool [61].

**Figure 19:**
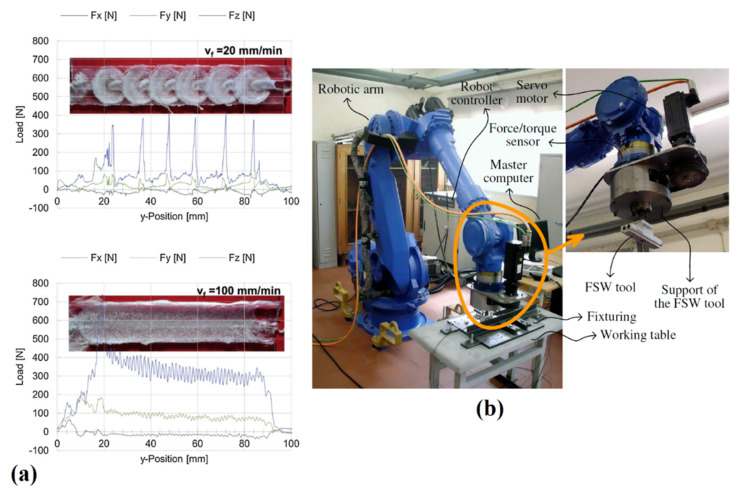
Load measurements during LFSW of (**a**) PC at different V [15] and (**b**) force control robotic system for LFSW of polymeric materials [62].

**Figure 20:**
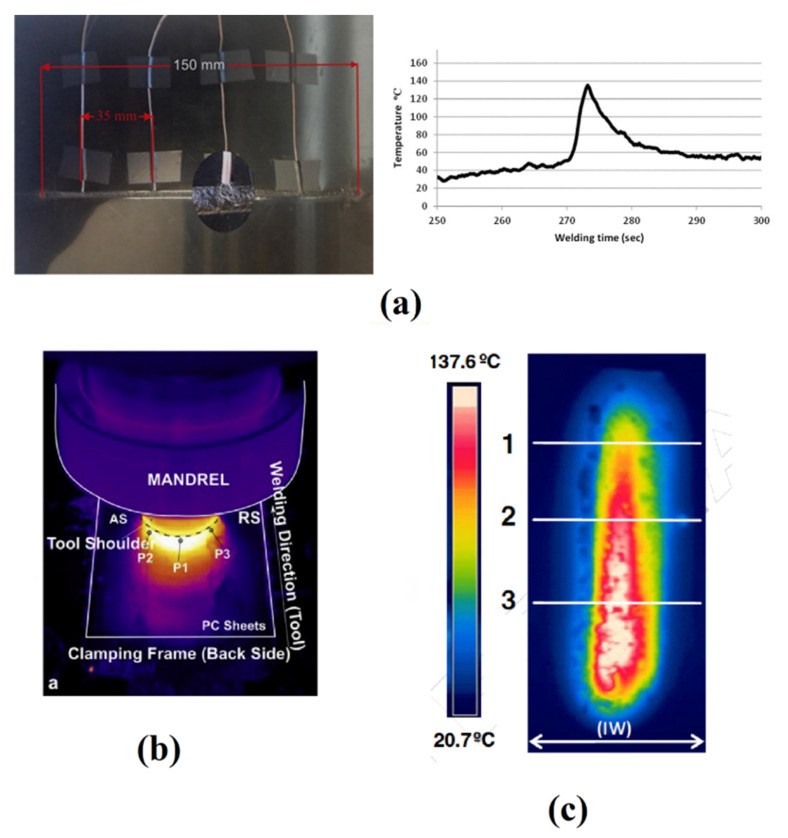
(**a**) K-type thermocouple used for recording temperature during LFSW of HMW-PE and its result [63], infrared (IR) camera picture of heat flux on surface of (**b**) PC [15] and (**c**) PMMA [64] during LFSW.

For the data analysis, the acquired signals are separated to different signals including signals of transverse direction (Fx), welding direction (Fy), and axial load (Fz). During LFSW of HMWPE, Eslami et al. [46] observed that the axial load raises upon the deformation of the weld nugget. After stabilizing and before welding, the frictional heat increases, while the value of Fz decreases. Axial forces beyond a certain value (950 N) resulted in the formation of thinner welds that affected the strength of the welds. The results of the study also indicated a relationship between Fx and Fy with the traverse speed and rotational velocity, respectively. At low welding speeds, the applied traversing force was low because the prolonged preheating leading to great flow stress reduction. At high rotational velocities, less torque was required during FSW, leading to lower lateral force. Lambiase et al. [15] measured the applied forces during FSW of PC. The load and temperature measurement trends were able to predict some material flow issues; for instance, when the load oscillated circular rifts were formed (Figure 19a). On the other hand, processing conditions characterized by more stable temperature and loads produced smoother as well as more regular material flow. It was also found that the measured loads depended on the tool welding speed. At low welding speeds, load fluctuation was increased due to enhanced hydrostatic pressure under the tool. Mendes et al. [62] developed a novel FSW robotic platform for welding of polymeric materials (Figure 12b). They showed that successful welding with an acceptable level of quality using a robotic FSW platform to control force and motion was feasible. Similarly, to load measurements, process temperature provides deep information concerning the process. This can be performed by means of contact (thermocouples) and non-contact (IR pyrometer or cameras) sensors. Temperature sensors can be inserted near the weld line to measure the profile upon processing. Eslami et al. [63] used K-type thermocouples beneath the weld nugget surface (as depicted in Figure 20) to determine the temperature variations at the bottom of HMPE sheets. During the experiments, some thermocouples were damaged because the thermocouples were involved in the material flow leading to the tearing of the contacts. In addition, when the tool passes too close to the thermocouple, it involves a further difficulty since the uncertainty of the measurement of the tool or that of the material.

The comparison between the recorded frictional heat and the HMPE melting temperature range confirms that friction stir welding of polymers is not an absolute solid-state welding process, but a mixture of molten materials with a relatively small amount of solid materials [63]. Thus, FSW develops more likely as a semisolid process. Thermocouples are generally embedded into the raw sheets at different distances from the weld seam. The results reported in [42] showed that preheating of the materials at the leading edge was almost negligible. The subsequent cooling rate was also slow. The heat was concentrated around the tool and heat flux on top surface of base materials was limited [65]. These observations confirmed that the temperature distribution during LFSW of polymers is much different as compared to that observed in metals. Alternatively, the temperature distribution and variation with time can be measured by means of non-contact techniques such as IR camera. This technique has some limitation that owes to the emissivity of polymers and acquisition rate. The distance and angle of camera to the joint line also affect the results [64]. These factors cause difficulties to calibrate and maybe is not appropriate for all polymers.

However, when applicable it provides much information about the process and temperature evolution. Compared to temperature measurements performed by means of thermocouples, IR measurements are characterized by shorter response time. In addition, IR cameras enable to measure the temperature of each pixel. This enable to produce a detailed map of temperature around the tool and its evolution during the process. Figure 20 shows the thermal profile around the tool measured by IR camera for PC and PMMA joints.

### 3.12. Special Tooling

Due to the different behavior of polymers during LFSW as compared to metals, different tools have been developed to improve the material flow and formation of defect free joints [66]. A comprehensive review of such developments has been reported in [6].

**Figure 21:**
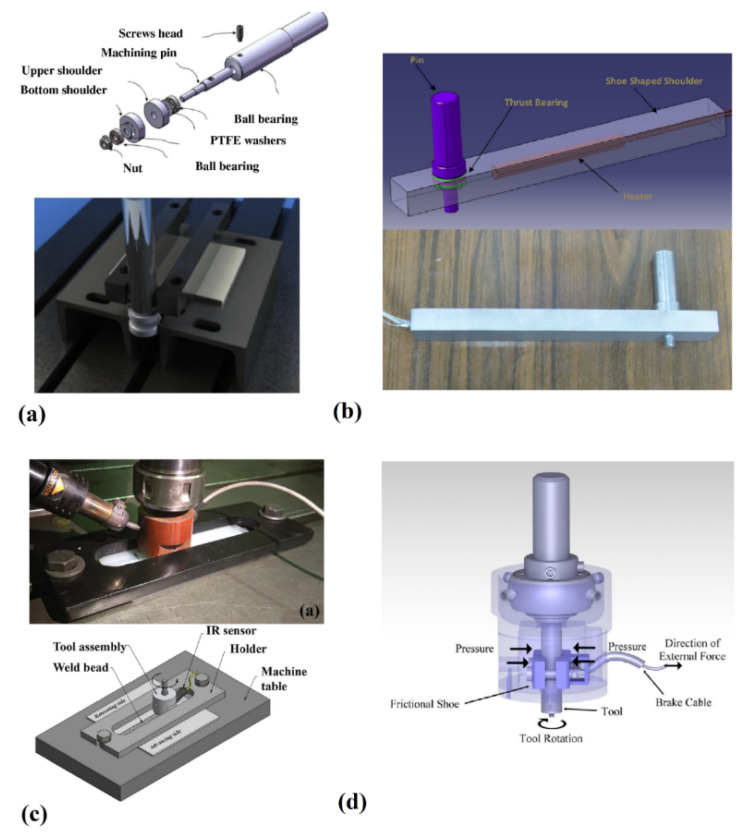
LFSW new designed tool used by (**a**) Pirizadeh et al. [66], (**b**) Azarsa and Mostafapour [67], (**c**) Moochani et al. [69], (**d**) Nath et al. [70].

Conventional LFSW tools generally lead to the formation of root defects in SZ that affects the mechanical properties of the welds. Pirizadeh et al. [66] used a tool with two shoulders which plunged the sheets along the weld seam from both the upper and lower side (Figure 21a). this enabled to reach a joint efficiency of almost 60%. Azarsa and Mostafapour [67] used a hot shoe (inherited from extrusion welding [3]). Here the shoulder does not rotate with the tool probe. A heater was located at the shoe shoulder to control the shoulder temperature on the leading and trailing edges of SZ (Figure 21b). This solution enabled the achievement of up to 96% of joint efficiency on HDPE. This tooling system was also adopted for ABS LFSW sheets by Bagheri et al. [47] The adoption of the hot shoe mainly lies in the possibility to fill the weld seam with the base material avoiding material ejection. This involves both an increase of the strength of the welds as well as a better weld appearance. Eslami et al. [48,68] used an LFSW tool with a small shoe to join PS and PP. The authors reported that the shoe shoulder enabled the production of stronger welds with good surface appearances. Moochani et al. [69] developed a compact tool assembly with auxiliary heating source (Figure 21c). They employed an IR sensor at the shoe to maintain the pin temperature constant without changing the other mechanical parameters. This enabled to achieve 96% of joint efficiency on welds made on PP. Nath et al. [70] used a self-heated tool for LFSW of PP (Figure 21d). A bearing was used to hold the tool shank in the center position of the tooling system and a skid rod was designed to fix the tool assembly to the FSW machine. This tool had a mechanism to control the axial pressure and surface contact of stationary shoulder with the surface of base material. The great advantage of this type of tool consisted into the tilting capability.

### 3.13. Simulation Modeling

Several analyses methods and design of experiments approaches have been used to understand the influence of process parameters on the material flow. Initial studies were mainly aimed at studying the feasibility of the process.

This was performed by establishing suitable process window and determining a strength-process parameters relationship. However, this approach has been rapidly substituted by more structured approaches aimed at understanding possible interactions among the process parameters. Then, Artificial Intelligence techniques were involved for the better description (and mapping) of processing conditions influence on the quality of the welds. However, a complete process understanding would require a deeper analysis of the material flow and possibly the availability to model its evolution during the process. Despite some studies concerning numerical modeling of friction stir welding of metals, only a couple of studies were performed [51,71]. These studies involved combined 3D non-linear heat transfer equations containing the internal rotational heat source, the momentum conservation principle, and temperature-dependent material properties [29,52,72,73] to simulate LFSW of PMMA and PC. Some assumptions were applied to simplify the governing equations for faster solving without affecting the accuracy, including:

- Base material supposed non-Newtonian fluid with visco-plastic flow behavior;
- Eulerian solution and adaptive meshing;
- Crystal structure changing during LFSW process was ignored.

**Figure 22:**
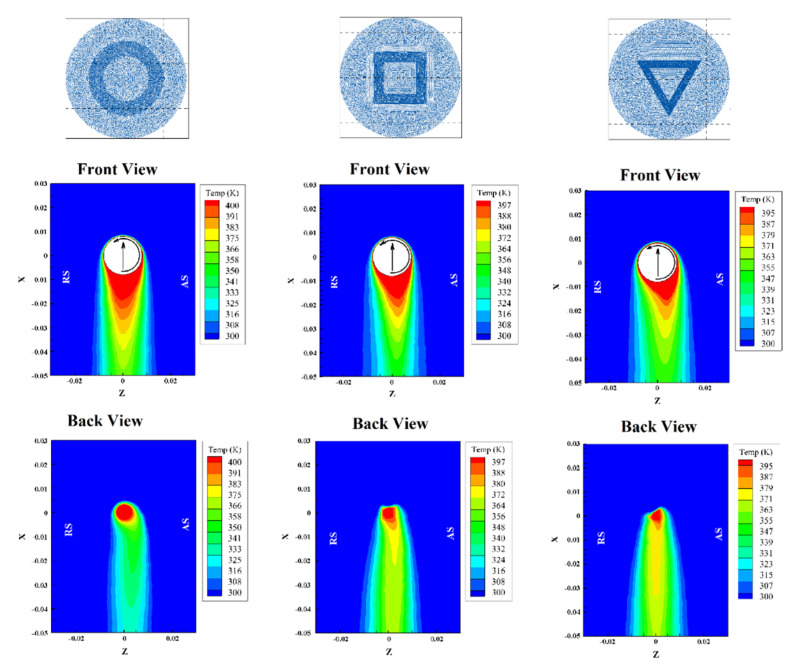
LFSW pin profile meshed and simulation results of heat flux on top and back side of PMMA sheet [72].

The results of simulations indicated that the peak temperature at the weld seam was produced at advancing side. The pressure gradient development controlled the formation of voids in SZ and root defects (e.g., like planar cracks). It was also found that the materials flow and inter-mixing during sting action was sensitive to the joint geometry. For instance, the heat generation and strain rate were more pronounced in LFSW of PMMA T-joints as compared with lap joint design [25]. The probe geometry greatly influenced the temperature if the material. Probes with sharp edges were not found appropriate for LFSW of semi-crystalline materials due to production of uneven pressure distribution at SZ [71]. Figure 22 shows simulation results determining heat flux on top and bottom surface of PMMA during LFSW. Although sound and useful results were obtained by simulation modeling with the assumptions, a combined computational fluid dynamic (CFD) and finite element (FE) model with temperature-pressure-thermal depend material properties is still missing.

## 4. Friction Spot Stir Welding

### 4.1. Description of the Main Phases

**Figure 23:**
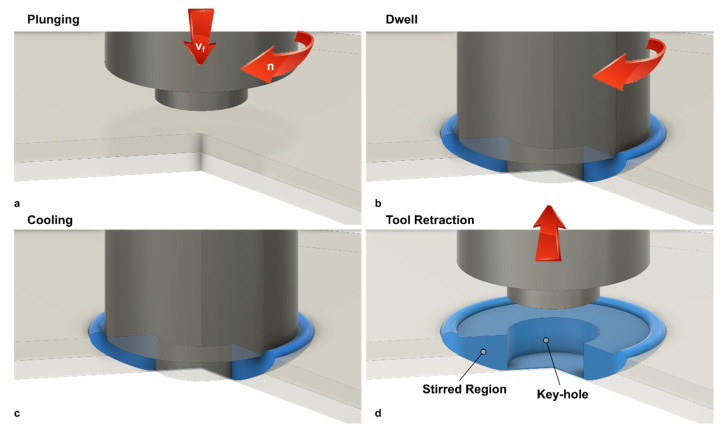
Main phases of FSSW development: (**a**) plunging; (**b**) dwell; (**c**) cooling and (**d**) tool retraction.

Friction stir welding can be performed to produce continuous and spot welds. In this latter case, the process is called friction spot stir welding (FSSW). During FSSW, the tool simply plunges the sheets placed in lap configuration upon a certain amount of material around the tool stir leading to the formation of the bond. Friction spot stir welding can be ideally subdivided into four phases, namely: plunging, dwell, cooling and punch retraction. During the plunging phase, the tool, which rotates at a prescribed speed, is plunged against the upper sheet and the probe tip reaches the lower sheet. The material moved by the tool probe extrudes vertically in form of ejected material. If the prescribed depth is reached, dwell starts and the plunge motion is stopped, while the tool continues to rotate. This phase is aimed at heating the material surrounding the tool probe where the connection between the sheets is formed. At the end of the dwell, cooling starts. This phase consists into stopping even the tool rotation. This enables the materials to cool down. This phase can be performed by holding the vertical position of tool, or under load control. This second way was demonstrated to have the capability to close the porosities and cavities that commonly form at the interface at the stirred region and surrounding material [74]. This can lead to superior mechanical behaviors of the welds. As the material has sufficiently cooled down, the tool is retracted. However, if this is performed prematurely, the retraction can lead to a tearing out effect of the material surrounding the tool [75], or even to material attached to the tool [76], as shown in Figure 23. A schematic of the main phases of FSSW is reported in Figure 23.

### 4.2. Material Flow

**Figure 24:**
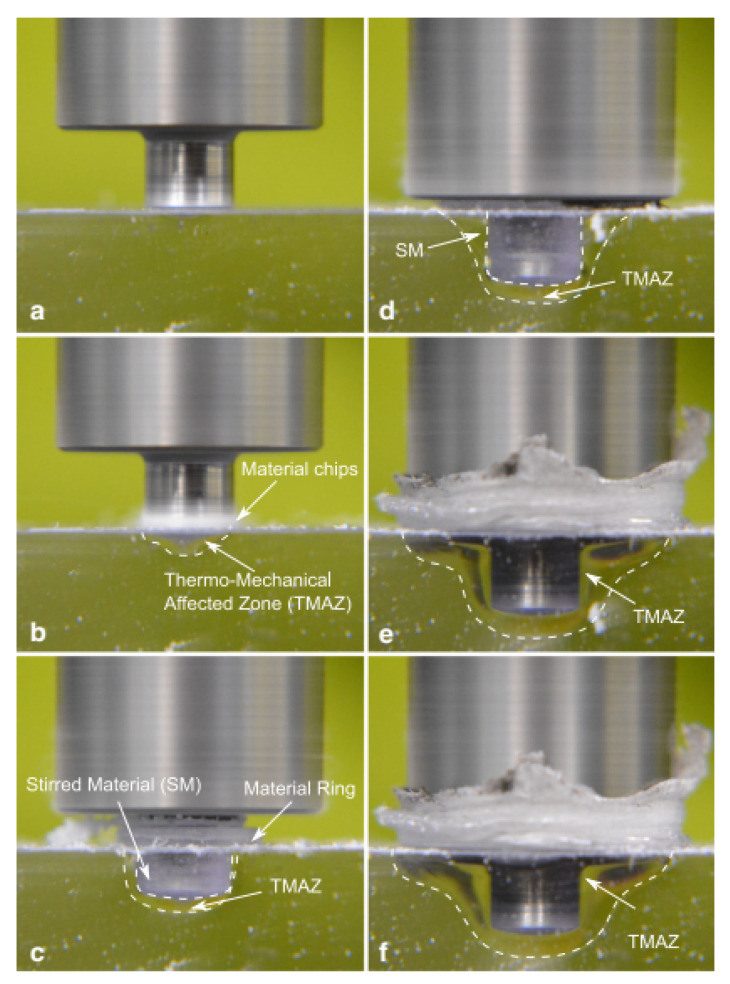
Material flow during FSSW of transparent polymers: (**a**) initial position; (**b**) formation of chips; (**c**) formation of TMAZ under the tool pin; (**d**) enlargement of the TAMZ; (**e**) deep plunging and (**f**) material after dwell.

Compared to metals, polymers are characterized by much lower thermal diffusivity and higher temperature sensitivity. This promotes the development of steep thermal gradients, which in turn determine regions with great differences in terms of mechanical behavior. Consequently, after a first contact between the tool and the upper layer, a thermo-mechanical affected zone immediately develops in the region underlying the tool probe, as shown in Figure 24b.

The material underlying the tool probe (TMAZ in Figure 24b,c) is characterized by a much higher temperature compared to the surrounding and underlying regions. Thus, it rapidly starts to reflow (reverse extrusion) vertically, as shown in Figure 24c. The TMAZ region extends progressively as the process proceeds, as the longer interaction time. This region also undergoes a drastic morphological change from a “U-shape” to “V-shape” as the tool shoulder approaches the upper surface of the upper sheet, as shown in Figure 24d. This owes to the contact of the tool shoulder with the polymer that leads to higher frictional heat and higher amount of material flow. The change to the “V-shape” of the TMAZ produces a dramatic increase of the mechanical behavior of the welds as it involves an increase in the extension of the weld area. Similarly, further plunging of the tool enables a downward shift of the TMAZ, which contributes to enlarge the weld area. During the dwell phase, the prolonged interaction time, which determines an increase of the frictional heat, also contributes to enlarge the welded region. This owes to heat diffusion effects that tend to increase the temperature of surrounding regions. The following cooling phase occurs. This is mainly aimed at enabling the tool retraction as soon as the material has restored its mechanical properties with a temperature well below the melting and softening temperatures of the polymer. Then, the tool is retracted. This comes with the formation of a blind hole in the joint (weld nugget) that is left by the tool probe.

**Figure 25:**
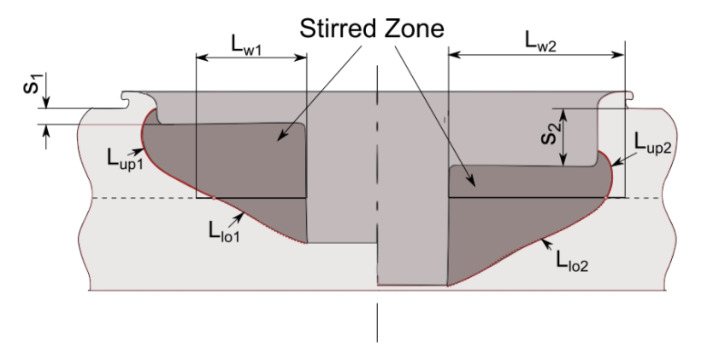
Effect of plunging depth on geometry of the weld [77].

As a result, the geometry of the friction spot stir welds can be characterized by the weld nugget, the stirred zone and the material reflow. This last feature is generally removed by common machining operations such as milling or drilling. On the other hand, the geometry and dimension of the weld nugget and stirred material determine the mechanical behavior of the welds. The characteristic dimensions of friction spot stir welds are reported in Figure 25.

### 4.3. Morphology of the Welds and Quality Assessment

To better comprehend the influence of the process parameters on the mechanical behavior of the welds, a deep analysis of the morphology of the joints and its influence on the failure mechanisms is required.

**Figure 26:**
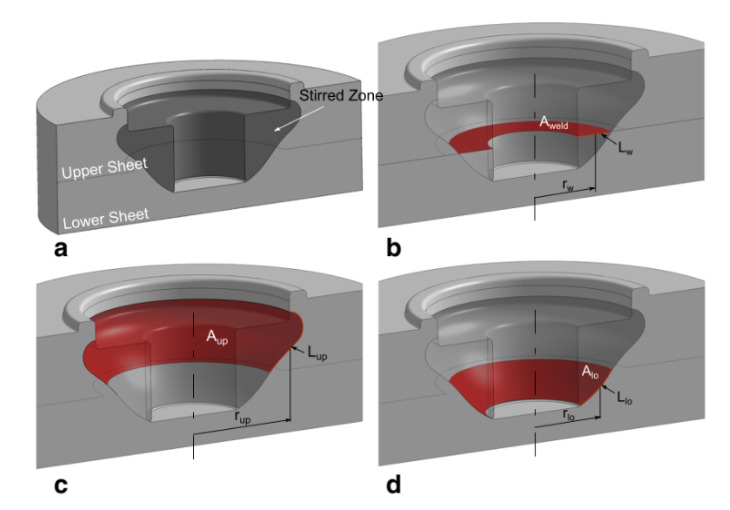
Characteristic interface areas of friction stir spot welds [74]: (**a**) stirred zone; (**b**) Aweld; (**c**) Aup and (**d**) Alow.

**Figure 27:**
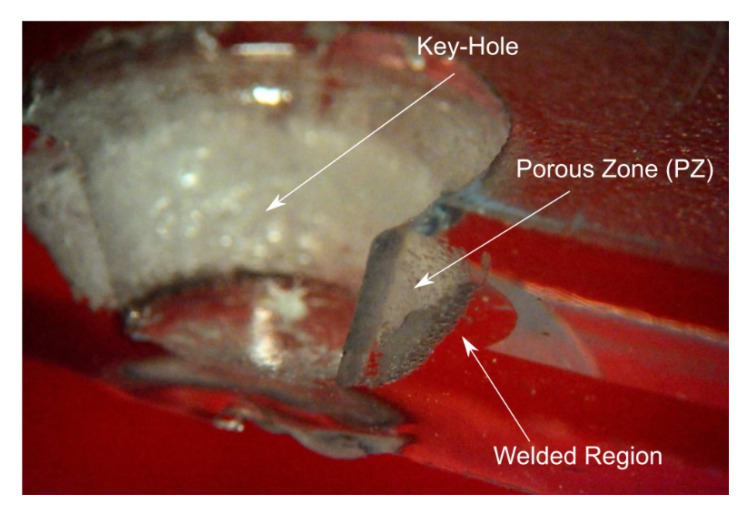
Presence of porous region at the interfaces between the stirred region and upper and lower sheets [77].

Figure 26a schematized an FSS weld as the connection of three regions: the upper sheet, the lower sheet and the stirred region. Although the upper and lower sheets are characterized by thermo-mechanical affected characteristics, they show their original compactness. On the one hand, the stirred region is often characterized by a certain porosity. This is mainly due to air trapped within the material during the process, as well as moisture of the original material in case of highly hygroscopic materials [78]. On the other hand, at the boundary of the stirred region with both the upper and lower sheets, bubbles and porosities can be highly concentrated. This is due to the thermal shrinkage of the material during the cooling phase. Steep temperature gradients develop between the stirred region and surrounding areas owing to the low thermal diffusion of polymers. Thus, the stirred region is at much higher temperature; consequently, during the cooling phase, the stirred region shrinks more than the upper and the lower sheets. This may cause the formation of a porous interface between the stirred region and the surrounding areas, as shown in Figure 27.

The failure of the FSS welds may occur in different ways, namely:

- Brittle fracture in the upper sheet (M1);
- Brittle fracture in the lower sheet (M2);
- Separation of the SZ from the lower sheet (M3);
- Separation of the SZ from the upper sheet (M4);
- Shear fracture in the SZ (M5).

**Figure 28:**
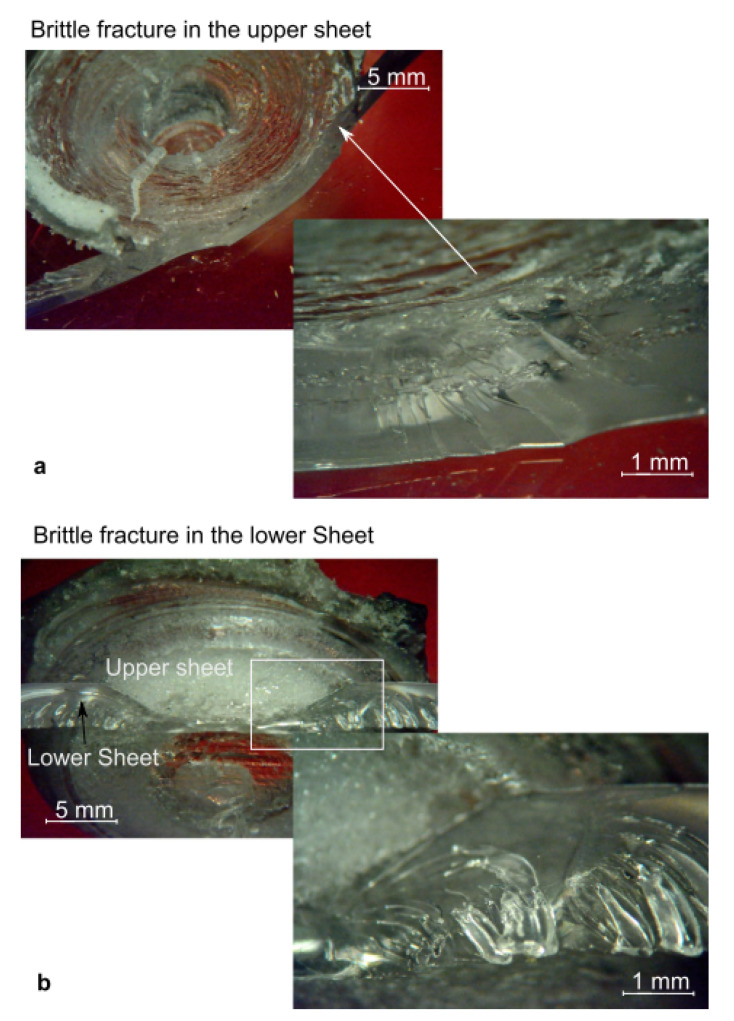
Fracture surfaces characterized by brittle failure [77]: (**a**) upper sheet and (**b**) lower sheet.

**Figure 29:**
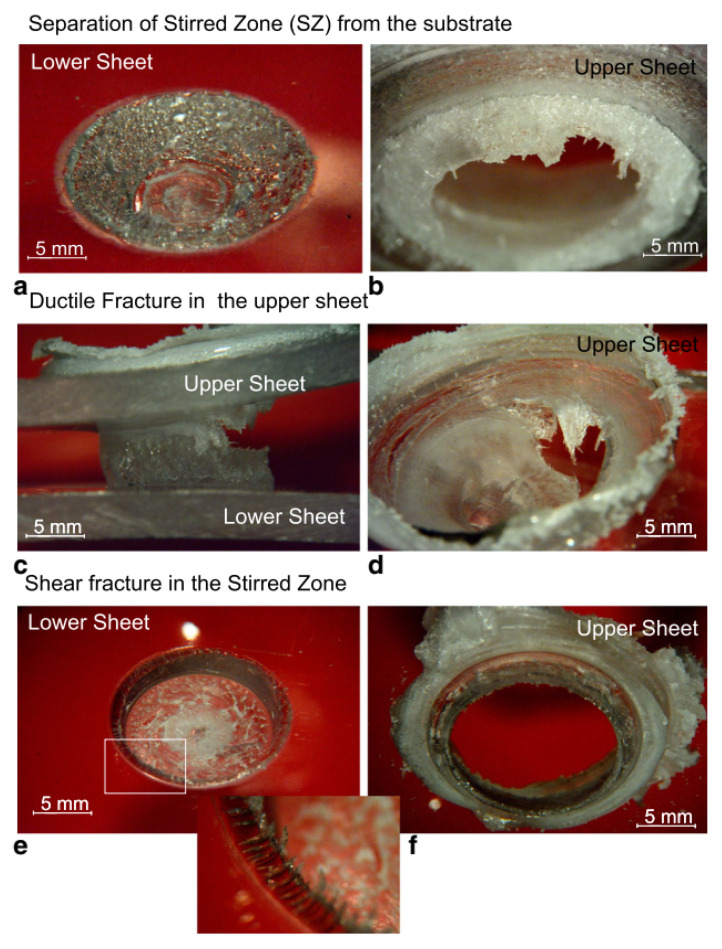
Typical failure modes of friction stir spot welds [77]. Separation of the SZ from the substrate in (**a**) lower sheet and (**b**) upper sheet. Ductile fracture in the upper sheet (**c**) side view and (**d**) upper view. (**e**,**f**) shear fracture in the stirred zone.

The fracture M1 and M2 developed almost instantly as the peak load was reached. These joints are usually characterized by a low absorbed energy but also high strength. Here the fracture develops almost instantly as a critical value of the stress (that is lower than the strength of the base material) is reached. The occurrence of the fracture owes to the presence of the porosities and the residual stress developing during the cooling phase and the high temperature gradients. The brittle behavior of such failure is evident from the fracture surfaces reported in Figure 28. On the other hand, because of the above-mentioned porous interface that may originate between the stirred region and surrounding material, failure M3 and M4 may develop. In these welds, the presence of the porosities reduces the contact area, acts as a stress raiser and induce the premature failure of the welds, as shown in Figure 29a–d. The welds characterized by a small stirred zone and especially a thin neck in correspondence of the separation line between the sheets, generally fail by shear failure, as depicted in Figure 29e–f. Here, the shear force concentrates in a small region.

The occurrence of one or another failure mode, is due to different physical phenomena, as well as the morphology and geometrical aspects. The formation of voids and porous interlayer between the stirred region and the base materials is often cause of Mode M3 or M4 failures. In addition, the presence of such defects within the stirred region may drive to shear failure (M5). However, geometrical aspects also play a great influence.

Lambiase et al. [74] analyzed the characteristic load bearing areas of friction spot stir welds, to better understand the influence of the optimal plunging depth. In failure modes M3 and M4, the stirred region behaves as an almost rigid body that progressively separates from the lower and upper sheets, respectively. The fracture originates at the boundary of the stirred region and propagates along the border with the base material. Thus, welds characterized by a large interface area between the stirred region and the upper sheet A_up_ (depicted in Figure 26c) and a low value of the interface area between the SR and the bottom sheet A_low_ (depicted in Figure 26d) are prone to fail by separation with the bottom sheet. Similarly, welds characterized by a small area A_weld_ (shown in Figure 26b) are generally fail by shear failure (M5). The quality of FSS welds and particularly the mechanical behavior depends on the material stirring quality as well as the presence of process induces defects. These defects can be thus classified in:

- Geometrical: incorrect choice of the plunging depth and/or tool dimension, shape of the tool probe;
- Morphological: formation of porosities, cavities. These depends on the selection of process parameters such as process speed, length of the dwell time, waiting time, load/displacement control, etc.
- Residual stress: thermal shrinkage leading to residual stress and formation of voids and cavities.

### 4.4. Effect of Process Parameters

**Figure 30:**
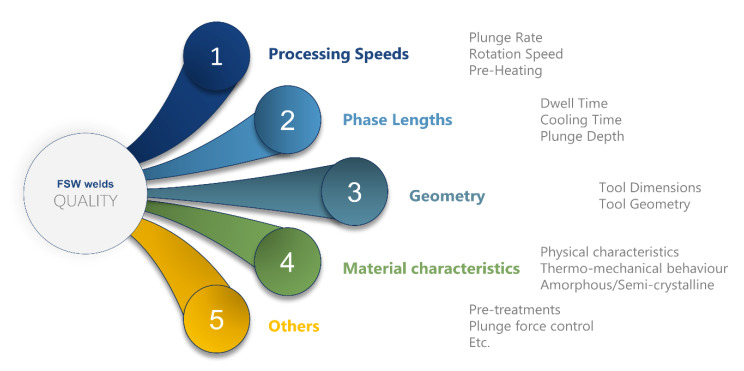
Factors influencing the quality of the welds.

The morphology of the welds is determined by several process parameters, which can be classified into: processing speeds, phases length and geometrical aspects, as summarized in Figure 30, and Table 5.

Before treating in detail the influence of the process parameters on the quality, morphology and mechanical behavior of friction spot stir welds, it must be noted that in some cases, there is not a full agreement among the results from different researches. This can be due by several causes, including adoption of different processing windows, materials, analysis methods, as well as depth of analysis. In addition, a further cause of misalignment concerning the influence of a given process parameter on the strength (especially for FSSW of polymers) is also represented by the relatively limited number of papers that have dealt with that process parameter. This makes it even more difficult to generalize the achievements of a certain paper outside the processing window analyzed in that paper.

**Figure 31:**
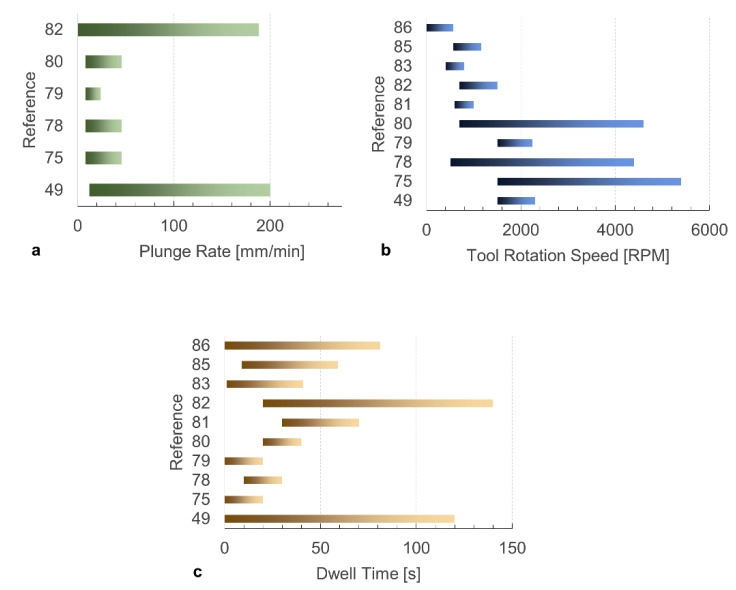
Processing windows analyzed in different papers concerning FSSW of polymers. Effect of (**a**) plunge rate; (**b**) tool rotation speed and (**c**) dwell time.

Figure 31 compares the ranges analyzed in different references for the plunging rate, tool rotation speed and dwell time. Obviously, a direct comparison would be inappropriate as different equipment’s (e.g., tool dimensions, machine capabilities, etc.), and materials are involved in these works. Nevertheless, a graphical representation of these ranges may provide qualitative insights into the processing windows studied. Figure 31a indicates a general consistency among the ranges studied for the plunging rate, the most studied plunge rate ranged between 20 and 40 mm/min. On the other hand, two separate windows can be identified, as shown in Figure 31b, pertaining to low (up to 500–2000 RPM) and high tool rotation speeds (2000–5500 RPM). Finally, three ranges for the dwell time were identified, as shown in Figure 31c: short (up to 20 s), medium (from 20 to 60 s) and long (up to 150 s) dwell time. It is worth noting that the papers involving high ranges of tool rotation speed [69,72,78], also involved short dwell time. This is not coincidental. Indeed, the frictional power is proportional to the tool rotation speed. Therefore, high tool rotation speed involves higher amount of frictional heat and consequently it demands lower dwell time to achieve the joints formation. Nevertheless, the adoption of low or high tool rotation speeds, which come with different power levels, may induce significant differences in temperature distributions. Since the low thermal diffusivity of polymers, the adoption of processing conditions involving high power are expected to produce steeper temperature gradients as those produced by processing conditions involving lower power amounts.

### 4.5. Plunge Rate

**Figure 32:**
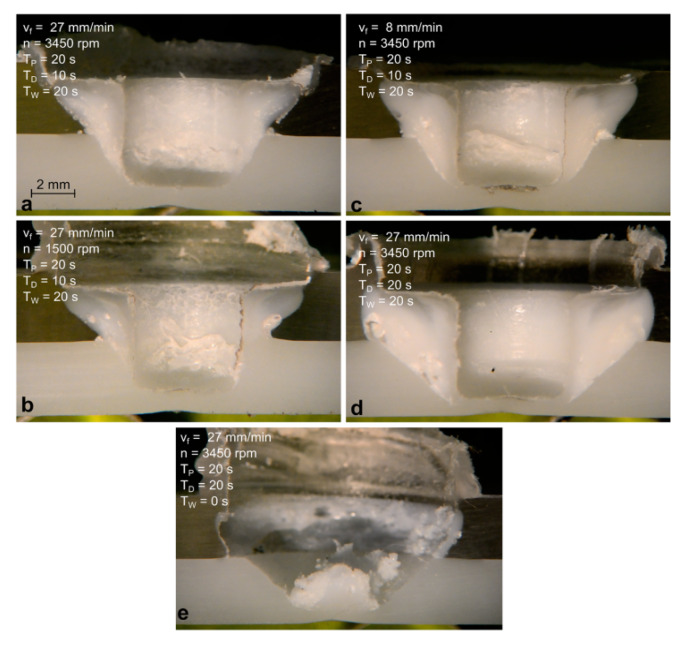
Influence of process parameters on welds morphology [78]. (**a**) reference condition; (**b**) reduction of tool rotation speed; (**c**) reduction of plunging speed; (**d**) increase of dwell time and (**e**) reduction of the waiting time.

The results reported by Yan et al. [49], indicated the existence of an optimal value of the plunge rate. The authors asserted that extremely low values of this parameter may affect the strength of the welds. This was addressed to defect formation, water absorption, molecular weight reduction, or residual stress generation. On the other hand, as reported by Lambiase et al. [78], extremely high values of the plunge rate may be also detrimental. The adoption of low values of the plunge rate involve short interaction time, lower weld dimensions and worse stirring homogenization and material heating that resulted in lower load bearing. This can be observed by comparing Figure 32a,b.

### 4.6. Rotation Speed

The influence of the tool rotation speed (commonly indicated with ω or n) has been investigated in several studies. The results do not fully agree about the effect of this process parameter. The results reported in [78,81] indicated a minor influence of n; on the other hand, other studies [82] determined the existence of an optimal value of the tool rotation speed that maximized the strength of the welds. Other studies determined a decreasing strength of the welds with the tool rotation speed [49]. It must be noted that the frictional heat produced by the tool is proportional to the tool rotation speed. Thus, higher values of n involve higher heating. However, because of the strong sensitivity of the mechanical behavior of the polymers by the temperature (especially close to the glass transition temperature), a non-linear relationship between the frictional heat and the tool rotation speed exists. Indeed, as the material is heated over the T_g_, the flow stress and the Young modulus drop. This comes with a steep reduction of the contact pressure and consequently the frictional heat. Thus, the difference among the abovementioned studies can be addressed to multiple reasons, including:

- The adoption of different ranges;
- The adoption of different tool dimensions;
- The investigation of different materials, which are characterized by different T_g_ and softening/melting points;
- The temperature reached during the process.

### 4.7. Pre-Heating Time

The results reported in [75,78,80] indicated that this parameter has negligible effect on the quality of the joints. The studies indicated that, the addition of the pre-heating phase while joining polymers provides a local heating of the material. Only the material underlying the tool probe is heated, while no significant effect develops in the surrounding regions. This was due to the poor thermal diffusivity of polymers. Consequently, the dimension of the welds is not modified as well as the strength.

### 4.8. Dwell Time

This parameter contributes to determine the amount of frictional heat supplied to the weld when the plunging depth is reached. During this phase, the heat enlarges the weld dimension. Thus, longer dwell time involve larger welds and consequently, higher load bearing, as can be observed by comparing Figure 32a,d reported in [78]. However, extremely high values of dwell time may come with detrimental effect. Indeed, as reported in [49], the adoption of extremely long dwell time may affect the strength of the welds. This was addressed to additional material extrusion and the generation of cavity as well as molecular weight decrease due to mechanical scission of molecular chains.

### 4.9. Cooling Time

**Figure 33:**
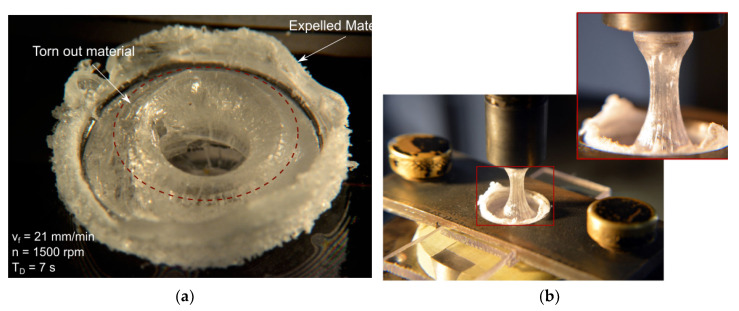
(**a**) Tearing out effect [75] and (**b**) material attached to the tool owing to highly insufficient waiting time [76].

At the end of the dwell phase, the material of the stirred region is under a rubber or pasty state. Thus, sufficient cooling time is required to prevent the tear of the SR by means of the tool tip, as shown in Figure 32e and Figure 33. Because of the poor thermal diffusivity of the polymers, the cooling of the stirred region requires a certain time. This depends on the peak temperature reached during the process, the dimension of the stirred region, as well as the thermal properties of the materials involved (the glass transition temperature). During this phase, the tool exerts a double function. Indeed, it applies a certain pressure on the contact surfaces with the stirred region. In addition, it drains heat from the weld enabling a faster cooling. The above considerations would drive towards a temperature-based trigger for tool retraction. As soon as the temperature drops under a given temperature (depending on the thermal characteristic of the polymer), the tool can be immediately retracted. This would involve obvious advantages in terms of process efficiency and productivity. However, this is hindered by the difficulty to measure the temperature of the stirred region during the process.

### 4.10. Plunge Depth

**Figure 34:**
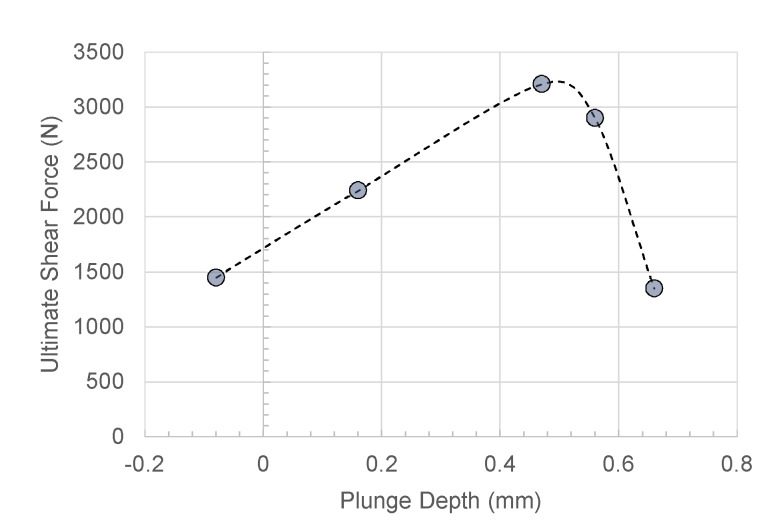
Influence of the penetration depth on the ultimate shear force (data from [77]).

The plunge depth directly affects the geometry and dimension of the characteristic regions of welds made by FSSW. Increasing the penetration depth (s), the area Aup (and Lup) decreases while the welds area Aweld and Alow increase, as shown in Figure 25. As expected, low penetration depth involved low strength as the weld area was very restricted. On the other hand, extremely high values of the tool plunge depth involved significant reduction of the Aup, which led to separation of the SR from the upper sheet. This condition also led to extremely low values of the ultimate shear force, as shown in Figure 34. The highest shear force found under intermediate values of the penetration depth.

### 4.11. Tool Shoulder and Probe Diameter

The results reported in [77] indicated that, the Ultimate Tensile Stress UTS of the welds increased when larger tool shoulder diameters were adopted and decreased with the tool probe diameter. This was due to the dimension of the stirred region and the characteristic areas. An increase of the tool shoulder diameter D comes with larger values of all the characteristic areas, Aup, Alow and Aweld. On the other hand, the dimension of the tool probe affected these areas and induced the reduction of the UTS of the welds. The selection of the proper processing conditions enabled to achieve a joint strength of 88% in the case of polycarbonate.

### 4.12. Geometry of the Tool Probe

Being a thermo-mechanical process based on the stirring of the two materials, the geometry of the tool probe strongly influences the strength of the welds. Bilici and Yükler [86] firstly investigated the influence of the tool pin on the mechanical behavior of FSS welds. The authors compared six different geometries, namely: cylindrical (SC), tapered cylindrical (TC), threaded cylindrical (TH), square (SQ), triangular (TR) and hexagonal (HG).

**Figure 35:**
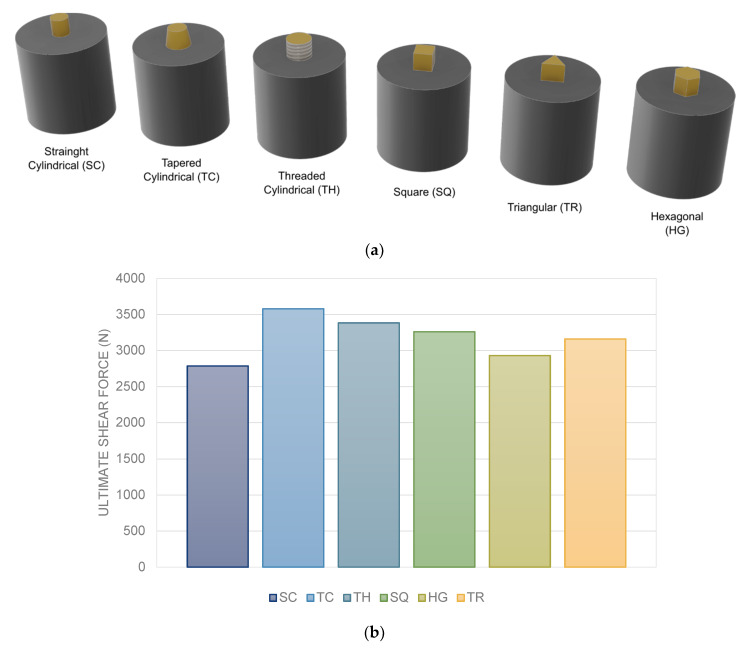
Influence of the tool probe shape on the mechanical behavior of the welds (data extracted from [86]). (**a**) pin shapes and (**b**) effect of pin shape on the UTS of the joint.

**Figure 36:**
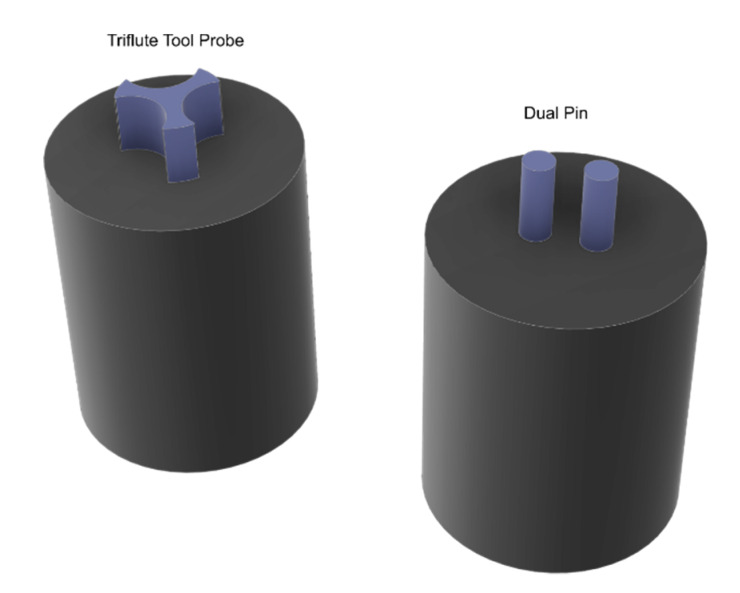
Schematic of triflute and dual pin used for FSSW of polymers.

The results reported in Figure 35 indicated that, the adoption of a threaded cylindrical probe enabled an increase of the ultimate shear force by 28% as compared to the welds made by a straight cylindrical shape, using the identical process parameters. This was addressed to a better material flow. The probe profile is also responsible of the weld nugget geometry and dimension after the tool retraction from the welds. Thus, this portion of material does not provide any load bearing capability. This idea drove further analysis concerning more exotic probe shapes. Yan et al. [85] investigated the adoption of a triflute tool probe to reduce the dimension of the weld nugget and reduce the amount of material ejected from the weld. This tool enabled significant increase in the welds force as the larger welds area. The authors also performed an experimental work concerning a double pin tool, which also enabled higher strength of the welds. The schematics of these probes are depicted in Figure 36.

### 4.13. Effect of Plunging Force

**Figure 37:**
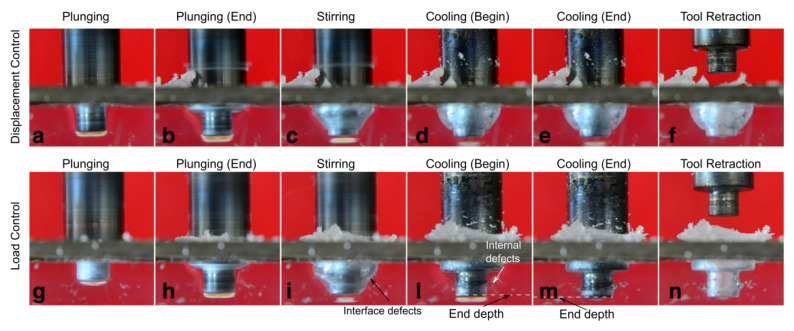
Effect of load control during cooling phase [74]. Sequence of processing phases under (**a**–**f**) displacement control and (**g**–**n**) load control.

Most of the papers concerning friction spot stir welding of polymers involved a displacement control during the entire welding process. This was due to a much simpler configuration of the machines adopted. However, this also involved the formation of a porous region surrounding the stirred zone. This region was characterized by the presence of large bubbles owing to moisture absorbed by the polymer, air trapped during the process as well as thermal shrinkage during the cooling phase. The presence of these bubbles dramatically affected the mechanical behavior of the welds as they reduced the load bearing are and acted as stress raisers. The sequence of the material flow during friction spot stir welding of transparent polycarbonate is reported in Figure 37a–f, as reported in [74]. Here, the formation of the bubbles between the stirred and surrounding regions is observed at the beginning of the dwell phase. During the plunging phase, the axial load exerts a compressive stress that hinders the bubble formation. However, as the tool motion is stopped, the axial load drops leading to easier formation of the bubbles.

To overcome the presence of this porous region, Lambiase et al. [74] involved load control during the cooling phase, as show in Figure 37g–n. This acted as compressive stress that enabled the closure of the porosities and cavities formed at the begin of the dwell phase. The results reported by the authors were extremely promising; indeed, the welds made under load control reached a joint efficiency of 99%.

## 5. Conclusions and Future Perspectives

Semisolid state joining processes of polymers and polymer composites are achieving great attention owing to the possibilities of producing welds with high strength, high automatization and low energy demand. These processes can be performed by means of CNC machines and robotic systems that enable the joining of dissimilar materials. Preliminary studies have been conducted to determine the suitability of linear and spot FSW of polymers. Then, following studies have treated more in depth the specific needs of FSW and FSSW when applied to polymeric materials instead of metals. This work attempted to describe with a structured approach the main phases of FSW and FSSW processes, the material flow that determined the morphology of the welds and the influence of the process parameters on the quality of the welds. Finally, recent achievements concerning process monitoring, special tooling adopted for polymers and simulation were discussed.

The analysis of literature indicated the lack of comprehensive data about FSW of all polymers. Many polymers, that have great engineering relevancy e.g., some high weight polymers PET, PBT, PMO, PEEK, LCP or PPS, have not yet studied. In addition, in many cases, the relationship between the characteristics of the polymer (chemical and physical behavior) and the processing conditions, is still unknown. A multidisciplinary approach involving different profiles including mechanical, processing, materials and chemistry engineers, would be beneficial to fully understand the polymer changes during semisolid state joining. Although sound processing windows have been determined for different polymers, a clear understanding of the influence of the material nature on the material flow is still missing, e.g., how amorphous and semi-crystalline polymers behave and how to modify the joining process based on polymer crystallinity. Long-term material behavior, relationship between shrinkage properties and process parameters, formability of FSWed polymeric structures, dynamic response of welded structures as well as fatigue properties of FSWed polymeric structures are still unknown.

The bibliographical analysis highlighted several aspects of the research carried out to date. For the linear friction stir welding, a considerable number of works have been carried out (although of some orders of magnitude smaller than the FSW of metals). This has made it possible to better focus the main problems of the process (e.g., excessive softening of the material under the rotating tool with expulsion of the same) and the identification of possible solutions (such as the use of hot shoe borrowed from the extrusion welding process). On the contrary, in the case of the FSSW, the small number of existing articles and especially the unevenness in the technological windows examined, indicate a lower maturity of the process and the need for greater efforts. From the industrial point of view, the adoption of one process with respect to another, as well as the sizing of a machine depend on both qualitative (mechanical characteristics, weld aesthetics) and productive factors (cycle time, process forces and energy). The bibliography has shown a greater attention to the qualitative aspects of welds, while only few papers analyzed the forces involved, the necessary powers and the welding energies. These aspects are fundamental to the sizing of the machines and to make a direct comparison with the costs (fixed and variable) of other welding processes.

**Figure 38:**
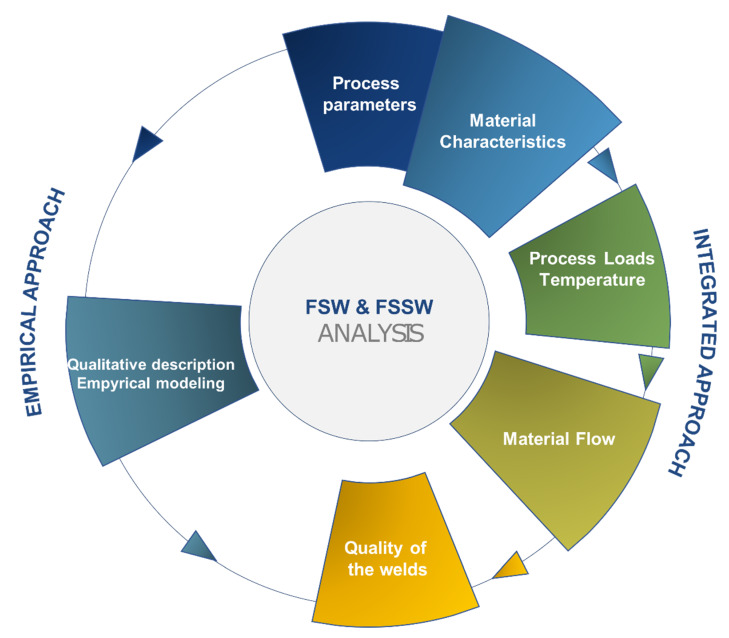
Comparison between empirical modeling approach vs. integrated approach.

Future studies concerning the friction stir welding processes of polymers should focus on the missing areas of knowledge and should also be concerned with changes of approach. Experimental studies should be performed more comprehensively, and not be limited to the description of the processing conditions–quality relationship. Rather, a more in-depth analysis should be conducted to discover the relationship between process conditions, the material flow and temperature distribution and the quality of the joints, as described in Figure 38.

## 6. Industry 4.0

**Figure 39:**
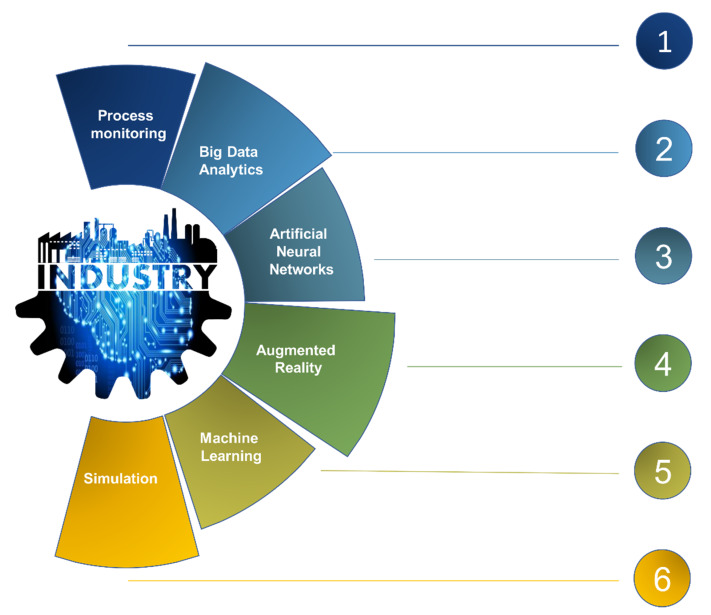
Industry 4.0 tools for FSW and FSSW processes understanding, automation, and optimization.

The availability of new technologies characterizing Industry 4.0 can allow to rapidly fill this lack of knowledge. Some efforts have been spent in recent papers concerning process monitoring (1) with the adoption of instrumented equipment (loads, torque, energy absorption, temperature measurement). This has enabled a deeper understanding of the influence of process parameters on the material flow. Different techniques (shown in Figure 39) pertaining to “Industry 4.0” are expected to provide a significant contribute for a deeper FSW and FSSW process understanding as well as optimization, and automation.

Big Data analytics (2) performed on processing signals acquired during the welding process could be used to determine, predict and eventually avoid process conditions that may lead to quality issues. To this end, artificial neural networks (3) and deep learning would represent promising tools for analysis of time series (for the analysis of processing loads and temperature). Augmented Reality (4) could be implemented by the adoption of CCD sensors and even webcams to determine the onset of unproper processing conditions (for example, excess of material ejection of material circular rifts [15]). Furthermore, the integration of augmented reality with process monitoring, Big Data analytics and artificial neural networks could also clarify the relationship between processing loads (and temperature) with the onset of adverse phenomena (material reflow, etc.).

The adoption of machine learning (5) based techniques could be exploited to model the process, and eventually drive the process itself by performing an online adjustment of the process parameters. Finally, the development of simulation (numerical) models capable of embedding the temperature dependent state variations during the process would enable the description of the material flow and temperature distributions that develop during the FSW and FSSW processes. A robust model could be developed, to determine how friction behavior at the tool/polymer interface produces polymer sticking to the tool during the process. In addition, it could be adopted to discover chemical changes and better understanding of the carbon chain flow during FSW.

## Figures and Tables

**Table 1:** Type of mechanical fastening.

Addition of Inserts	Molded in Connections	Flow Joining-Plastic Deformation-Thermoforming
Screw, nut, bolts and washers	Molded-in threads	Stacking (Air, Ultrasonic, Vibration, Friction)
Expansion inserts	Molded in inserts	Hemming
Self-tapping inserts	Snap-fits	–
Ultrasonic insert	–	–
Rivets	–	–

**Table 2:** Pro and cons of mechanical fastening.

Advantages	Limits and Disadvantages
Simplicity	Stress concentration
Permanent or nonpermanent	Increase of weight
Possibly to be directly embedded into the mold (snap fits)	Costs
High mechanical behavior even at high temperatures	Visibility from one or both sides of the connector
Possibility to join materials with great differences	Many processes require pre-drilling

**Table 3:** Pro and cons of friction stir welding and friction spot stir welding.

Advantages	Limits and Disadvantages
Localized heating	Relatively high forces involved
Low energy requirement	Requires high stiffness of the equipment
High strength Reduced material distortion and residual Stress	The process can worsen the appearance (especially on correspondence contact surface with the tool shoulder) of the weld seam.
Possibility to join different materials	High investment costs
No surface pretreatment is required	–
Relatively high speed	–
Easiness of automation	–
Low process variability	–

**Table 4:** LFSW process parameters List.

Category	Process Parameter	Brief Description	References
Processing Speeds	Rotation Speed	Rotational speed of the LFSW tool during the welding process	[13,25,29,36,38,39,40,41,42,43,44,45,46,47,48,49]
	Welding Speed	Forward moving speed of the LFSW tool during the welding process along joint line	
Processing Variables	Plunge Depth	Final penetration depth of LFSW tool on top surface of base materials. During the welding process LFSW tool plunge depth remains constant while the tool continues to rotate	[29,36,42,49,50]
	Tilt Angle	Axial tilt of LFSW tool compare surface of base material normal axis. The tool tilt angle has negative amount toward LFSW tool forward moving direction	[28,38,39,40,41,42,45,50,51,52]
Geometry	Tool probe profile	Force applied during the dwell phase when load-control is involved in the process	[15,29,42,43,44,45,48,49,51,52]

**Table 5:** Classification of process parameters for FSSW.

Category	Process Parameter	Brief Description	References
Processing Speeds	Plunge Rate	Speed of the tool during the plunging phase upon reaching the penetration depth	[49,75,78,79,80]
	Rotation speed	Speed of the tool during the plunging and dwell phases	[49,79,81,82,83]
Phases Length	Pre-heating time	Period of material pre-heating by slight plunging of the tool over the upper sheet	[49,79,81,82,83]
	Dwell time	time elapsing since the tool has reached the final penetration depth and start of cooling. During this period, the tool plunge is stopped while the tool continues to rotate	[49,75,78,80,82]
	Cooling time	Period during which the tool is fully stopped (no plunging and rotation take place)	[49,81,82,83]
Geometry	Plunge depth	Axial displacement of the tool since the first contact with the upper sheet to the final position. This should be greater than the upper sheet thickness and lower than the sum of the sheet thicknesses	[74,76,77]
	Tool shoulder diameter	–	[74,76,77]
	Tool probe diameter	–	[76,77,84]
	Tool probe geometry	–	[76,77,84,85,86]
Others	Plunge force	Force applied during the dwell phase when load-control is involved in the process	[74]

## References

[1] Lambiase F., Ko D.-C. (2016). Feasibility of mechanical clinching for joining aluminum AA6082-T6 and Carbon Fiber Reinforced Polymer sheets. Mater. Des..

[2] Katayama S. (2013). Handbook of Laser Welding Technologies.

[3] Troughton M. (2008). Handbook of Plastics Joining: A Practical Guide.

[4] Camanho P.P., Tong L. (2011). Composite Joints and Connections. Principles, Modelling and Testing.

[5] Kiss Z., Czigány T. (2007). Applicability of friction stir welding in polymeric materials. Period. Polytech. Mech. Eng..

[6] Eslami S., Tavares P.J., Moreira P.M.G.P. (2016). Friction stir welding tooling for polymers: Review and prospects. Int. J. Adv. Manuf. Technol..

[7] Kumar R., Singh R., Ahuja I.P.S., Penna R., Feo L. (2018). Weldability of thermoplastic materials for friction stir welding—A state of art review and future applications. Compos. Part B Eng..

[8] Lambiase F., Ko D.C. (2017). Two-steps clinching of aluminum and Carbon Fiber Reinforced Polymer sheets. Compos. Struct..

[9] Lambiase F., Paoletti A., Di Ilio A. (2017). Advances in Mechanical Clinching: Employment of a Rotating Tool. Procedia Eng..

[10] Lambiase F., Paoletti A. (2018). Friction-assisted clinching of Aluminum and CFRP sheets. J. Manuf. Process..

[11] Lee C.-J., Shen G., Kim B.-M., Lambiase F., Ko D.-C. (2018). Analysis of Failure-Mode Dependent Joint Strength in Hole Clinching from the Aspects of Geometrical Interlocking Parameters. Metals.

[12] Derazkola H.A., Elyasi M. (2018). The influence of process parameters in friction stir welding of Al-Mg alloy and polycarbonate. J. Manuf. Process..

[13] Derazkola H.A., Khodabakhshi F., Simchi A. (2018). Friction-stir lap-joining of aluminium-magnesium/poly-methyl-methacrylate hybrid structures: Thermo-mechanical modelling and experimental feasibility study. Sci. Technol. Weld. Join..

[14] Lambiase F., Paoletti A., Di Ilio A. (2018). Forces and temperature variation during friction stir welding of aluminum alloy AA6082-T6. Int. J. Adv. Manuf. Technol..

[15] Lambiase F., Paoletti A., Grossi V., Di Ilio A. (2019). Analysis of loads, temperatures and welds morphology in FSW of polycarbonate. J. Mater. Process. Technol..

[16] Derazkola H.A., Simchi A. (2019). An investigation on the dissimilar friction stir welding of T-joints between AA5754 aluminum alloy and poly(methyl methacrylate). Thin-Walled Struct..

[17] Nandan R., DebRoy T., Bhadeshia H.K.D.H. (2008). Recent advances in friction-stir welding–Process, weldment structure and properties. Prog. Mater. Sci..

[18] Simar A., Bréchet Y., de Meester B., Denquin A., Gallais C., Pardoen T. (2012). Integrated modeling of friction stir welding of 6xxx series Al alloys: Process, microstructure and properties. Prog. Mater. Sci..

[19] He X., Gu F., Ball A. (2014). A review of numerical analysis of friction stir welding. Prog. Mater. Sci..

[20] Derazkola H.A., Khodabakhshi F. (2020). A novel fed friction-stir (FFS) technology for nanocomposite joining. Sci. Technol. Weld. Join..

[21] Elyasi M., Aghajani Derazkola H., Hosseinzadeh M. (2016). Investigations of tool tilt angle on properties friction stir welding of A441 AISI to AA1100 aluminium. Proc. Inst. Mech. Eng. Part B J. Eng. Manuf..

[22] Derazkola H.A., Aval H.J., Elyasi M. (2015). Analysis of process parameters effects on dissimilar friction stir welding of AA1100 and A441 AISI steel. Sci. Technol. Weld. Join..

[23] Derazkola H.A., Fard R.K., Khodabakhshi F. (2018). Effects of processing parameters on the characteristics of dissimilar friction-stir-welded joints between AA5058 aluminum alloy and PMMA polymer. Weld. World.

[24] Huang Y., Meng X., Xie Y., Wan L., Lv Z., Cao J., Feng J. (2018). Friction stir welding/processing of polymers and polymer matrix composites. Compos. Part A Appl. Sci. Manuf..

[25] Elyasi M., Derazkola H.A. (2018). Experimental and thermomechanical study on FSW of PMMA polymer T-joint. Int. J. Adv. Manuf. Technol..

[26] Moreno-Moreno M., Macea Romero Y., Rodríguez Zambrano H., Restrepo-Zapata N.C., Afonso C.R.M., Unfried-Silgado J. (2018). Mechanical and thermal properties of friction-stir welded joints of high density polyethylene using a non-rotational shoulder tool. Int. J. Adv. Manuf. Technol..

[27] Kumar S., Roy B.S. (2019). Novel study of joining of acrylonitrile butadiene styrene and polycarbonate plate by using friction stir welding with double-step shoulder. J. Manuf. Process..

[28] Arici A., Sinmazçelýk T. (2005). Effects of double passes of the tool on friction stir welding of polyethylene. J. Mater. Sci..

[29] Mosavvar A., Azdast T., Moradian M., Hasanzadeh R. (2019). Tensile properties of friction stir welding of thermoplastic pipes based on a novel designed mechanism. Weld. World.

[30] (2000). ASTM D 638. Standard Test Method for Tensile Properties of Plastics.

[31] Derazkola H.A., Simchi A. (2018). Effects of alumina nanoparticles on the microstructure, strength and wear resistance of poly(methyl methacrylate)-based nanocomposites prepared by friction stir processing. J. Mech. Behav. Biomed. Mater..

[32] Gao J., Cui X., Liu C., Shen Y. (2017). Application and exploration of friction stir welding/processing in plastics industry. Mater. Sci. Technol..

[33] (2000). ASTM D2240-Standard Test Method for Rubber Property—Durometer Hardness.

[34] Lambiase F., Grossi V., Paoletti A. (2019). Advanced mechanical characterization of friction stir welds made on polycarbonate. Int. J. Adv. Manuf. Technol..

[35] Vijendra B., Sharma A. (2015). Induction heated tool assisted friction-stir welding (i-FSW): A novel hybrid process for joining of thermoplastics. J. Manuf. Process..

[36] Nandhini R., Moorthy M.K., Muthukumaran S. (2017). Effect of Welding Parameters on Microstructure and Tensile Strength of Friction Stir Welded PA 6,6 Joints. Int. Polym. Process..

[37] Gao J., Shen Y., Zhang J., Xu H. (2014). Submerged friction stir weld of polyethylene sheets. J. Appl. Polym. Sci..

[38] Zafar A., Awang M., Khan S.R., Emamian S. (2016). Investigating Friction Stir Welding on Thick Nylon 6 Plates. Weld. Res..

[39] Saeedy S., Givi M.K.B. (2011). Investigation of the effects of critical process parameters of friction stir welding of polyethylene. Proc. Inst. Mech. Eng. Part B J. Eng. Manuf..

[40] Bozkurt Y. (2012). The optimization of friction stir welding process parameters to achieve maximum tensile strength in polyethylene sheets. Mater. Des..

[41] Jaiganesh V., Maruthu B., Gopinath E. (2014). Optimization of Process Parameters on Friction Stir Welding of High Density Polypropylene Plate. Procedia Eng..

[42] Derazkola H.A., Simchi A., Lambiase F. (2019). Friction stir welding of polycarbonate lap joints: Relationship between processing parameters and mechanical properties. Polym. Test..

[43] Rezaee Hajideh M., Farahani M., Alavi S.A.D., Molla Ramezani N. (2017). Investigation on the effects of tool geometry on the microstructure and the mechanical properties of dissimilar friction stir welded polyethylene and polypropylene sheets. J. Manuf. Process..

[44] Sahu S.K., Mishra D., Mahto R.P., Sharma V.M., Pal S.K., Pal K., Banerjee S., Dash P. (2018). Friction stir welding of polypropylene sheet. Eng. Sci. Technol. Int. J..

[45] Hoseinlaghab S., Mirjavadi S.S., Sadeghian N., Jalili I., Azarbarmas M., Besharati Givi M.K. (2015). Influences of welding parameters on the quality and creep properties of friction stir welded polyethylene plates. Mater. Des..

[46] Eslami S., Mourão L., Viriato N., Tavares P.J., Moreira P.M.G.P. (2018). Multi-axis force measurements of polymer friction stir welding. J. Mater. Process. Technol..

[47] Bagheri A., Azdast T., Doniavi A. (2013). An experimental study on mechanical properties of friction stir welded ABS sheets. Mater. Des..

[48] Eslami S., Ramos T., Tavares P.J., Moreira P.M.G.P. (2015). Shoulder design developments for FSW lap joints of dissimilar polymers. J. Manuf. Process..

[49] Yan Y., Shen Y., Zhang W., Guan W. (2017). Effects of friction stir spot welding parameters on morphology and mechanical property of modified cast nylon 6 joints produced by double-pin tool. Int. J. Adv. Manuf. Technol..

[50] Lambiase F., Grossi V., Paoletti A. (2020). Effect of tilt angle in FSW of polycarbonate sheets in butt configuration. Int. J. Adv. Manuf. Technol..

[51] Aghajani Derazkola H., Simchi A. (2018). Experimental and thermomechanical analysis of friction stir welding of poly(methyl methacrylate) sheets. Sci. Technol. Weld. Join..

[52] Eyvazian A., Hamouda A.M., Aghajani Derazkola H., Elyasi M. (2019). Study on the effects of tool tile angle, offset and plunge depth on friction stir welding of poly(methyl methacrylate) T-joint. Proc. Inst. Mech. Eng. Part B: J. Eng. Manuf..

[53] Kiss Z., Czigány T. (2012). Effect of welding parameters on the heat affected zone and the mechanical properties of friction stir welded poly(ethylene-terephthalate-glycol). J. Appl. Polym. Sci..

[54] Ülker A., Sayer S., Ceyhun V. (2016). Welding parameters and joint strength optimization during friction stir welding of high density polyethylene (HDPE) using the Taguchi method. Mater. Test..

[55] Arici A., Selale S. (2007). Effects of tool tilt angle on tensile strength and fracture locations of friction stir welding of polyethylene. Sci. Technol. Weld. Join..

[56] Lambiase F., Grossi V., Di Ilio A., Paoletti A. (2020). Feasibility of friction stir joining of polycarbonate to CFRP with thermosetting matrix. Int. J. Adv. Manuf. Technol..

[57] Sadeghian N., Besharati Givi M.K. (2015). Experimental optimization of the mechanical properties of friction stir welded Acrylonitrile Butadiene Styrene sheets. Mater. Des..

[58] Panneerselvam K., Lenin K. (2014). Joining of Nylon 6 plate by friction stir welding process using threaded pin profile. Mater. Des..

[59] Gao J., Shen Y., Xu H. (2015). Investigations for the mechanical, macro-, and microstructural analyses of dissimilar submerged friction stir welding of acrylonitrile butadiene styrene and polycarbonate sheets. Proc. Inst. Mech. Eng. Part B J. Eng. Manuf..

[60] Yan Y., Shen Y., Lan B., Gao J. (2017). Influences of friction stir welding parameters on morphology and tensile strength of high density polyethylene lap joints produced by double-pin tool. J. Manuf. Process..

[61] Trimble D., Monaghan J., O’Donnell G.E. (2012). Force generation during friction stir welding of AA2024-T3. Cirp Ann. Manuf. Technol..

[62] Mendes N., Neto P., Simão M.A., Loureiro A., Pires J.N. (2016). A novel friction stir welding robotic platform: Welding polymeric materials. Int. J. Adv. Manuf. Technol..

[63] Eslami S., Francisco Miranda J., Mourão L., Tavares P.J., Moreira P.M.G.P. (2018). Polyethylene friction stir welding parameter optimization and temperature characterization. Int. J. Adv. Manuf. Technol..

[64] Simões F., Rodrigues D.M. (2014). Material flow and thermo-mechanical conditions during Friction Stir Welding of polymers: Literature review, experimental results and empirical analysis. Mater. Des..

[65] Nandhini R., Moorthy M.K., Muthukumaran S., Kumaran S. (2019). Influence of process variables on the characteristics of friction-stir-welded polyamide 6,6 joints. Mater. Und Werkst..

[66] Pirizadeh M., Azdast T., Rash Ahmadi S., Mamaghani Shishavan S., Bagheri A. (2014). Friction stir welding of thermoplastics using a newly designed tool. Mater. Des. (1980–2015).

[67] Azarsa E., Mostafapour A. (2014). Experimental investigation on flexural behavior of friction stir welded high density polyethylene sheets. J. Manuf. Process..

[68] Eslami S., de Figueiredo M.A.V., Tavares P.J., Moreira P.M.G.P. (2018). Parameter optimisation of friction stir welded dissimilar polymers joints. Int. J. Adv. Manuf. Technol..

[69] Moochani A., Omidvar H., Ghaffarian S.R., Goushegir S.M. (2019). Friction stir welding of thermoplastics with a new heat-assisted tool design: mechanical properties and microstructure. Weld. World.

[70] Nath R.K., Maji P., Barma J.D. (2019). Development of a Self-Heated Friction Stir Welding tool for welding of polypropylene sheets. J. Braz. Soc. Mech. Sci. Eng..

[71] Derazkola H.A., Eyvazian A., Simchi A. (2020). Modeling and experimental validation of material flow during FSW of polycarbonate. Mater. Today Commun..

[72] Derazkola H.A., Simchi A. (2018). Experimental and thermomechanical analysis of the effect of tool pin profile on the friction stir welding of poly(methyl methacrylate) sheets. J. Manuf. Process..

[73] Mohammadi Kuhbanani H., Yasemi H., Aghajani Derazkola H. (2018). Effects of Tool Tilt Angle and Plunge Depth on Properties of Polycarbonate FSW Joint. J. Mod. Process. Manuf. Prod..

[74] Lambiase F., Paoletti A., Di Ilio A. (2017). Friction spot stir welding of polymers: control of plunging force. Int. J. Adv. Manuf. Technol..

[75] Paoletti A., Lambiase F., Di Ilio A. (2016). Analysis of forces and temperatures in friction spot stir welding of thermoplastic polymers. Int. J. Adv. Manuf. Technol..

[76] Lambiase F., Paoletti A., Di Ilio A. (2016). Effect of tool geometry on loads developing in friction stir spot welds of polycarbonate sheets. Int. J. Adv. Manuf. Technol..

[77] Lambiase F., Paoletti A., Di Ilio A. (2017). Effect of tool geometry on mechanical behavior of friction stir spot welds of polycarbonate sheets. Int. J. Adv. Manuf. Technol..

[78] Lambiase F., Paoletti A., Di Ilio A. (2015). Mechanical behaviour of friction stir spot welds of polycarbonate sheets. Int. J. Adv. Manuf. Technol..

[79] Dashatan S.H., Azdast T., Ahmadi S.R., Bagheri A. (2013). Friction stir spot welding of dissimilar polymethyl methacrylate and acrylonitrile butadiene styrene sheets. Mater. Des..

[80] Paoletti A., Lambiase F., Di Ilio A. (2015). Optimization of Friction Stir Welding of Thermoplastics. Procedia CIRP.

[81] Bilici M.K. (2012). Application of Taguchi approach to optimize friction stir spot welding parameters of polypropylene. Mater. Des..

[82] Memduh K. (2012). Friction stir spot welding parameters for polypropylene sheets. Sci. Res. Essays.

[83] Bilici M.K., Yükler A.İ., Kurtulmuş M. (2011). The optimization of welding parameters for friction stir spot welding of high density polyethylene sheets. Mater. Des..

[84] Yan Y., Shen Y., Hou W., Li J. (2018). Friction stir spot welding thin acrylonitrile butadiene styrene sheets using pinless tool. Int. J. Adv. Manuf. Technol..

[85] Yan Y., Shen Y., Zhang W., Hou W. (2018). Friction stir spot welding ABS using triflute-pin tool: Effect of process parameters on joint morphology, dimension and mechanical property. J. Manuf. Process..

[86] Bilici M.K., Yükler A.İ. (2012). Influence of tool geometry and process parameters on macrostructure and static strength in friction stir spot welded polyethylene sheets. Mater. Des..

